# Methodological Approaches to Assess Disordered Eating Behaviors Related to Gluten-Free Diet Management in Children and Adolescents with Celiac Disease: A Scoping Review

**DOI:** 10.3390/nu18111661

**Published:** 2026-05-22

**Authors:** Marina de Cesaro Schwantes, Rafaella Dusi, Rosa Harumi Uenishi, Camila dos Santos Ribeiro, Renata Puppin Zandonadi

**Affiliations:** 1 Department of Nutrition, Faculty of Health Sciences, Darcy Ribeiro University Campus, University of Brasília, Brasília 70910-900, Brazil; rafaella.dusi@gmail.com (R.D.); camilasribeiro15@gmail.com (C.d.S.R.); 2 Interdisciplinary Laboratory of Biosciences, Faculty of Medicine, Darcy Ribeiro University Campus, University of Brasília, Brasília 70910-900, Brazil; rosa.uenishi@gmail.com

**Keywords:** gluten-free diet, psychometrics, behavioral nutrition, disordered eating

## Abstract

**Objective:** This scoping review aimed to map the methodological approaches used to assess disordered eating attitudes and behaviors in children and adolescents with celiac disease (CD). **Methods:** This review followed the Joanna Briggs Institute scoping review methodology and the PRISMA-ScR guidelines, including studies of children and adolescents with CD that used methodological approaches to assess disordered eating attitudes and behaviors in the context of the gluten-free diet (GFD). No restrictions were applied regarding geography, language, or year. Searches were conducted across 10 electronic databases. Data were descriptively analyzed and presented in tables or diagrams, with a narrative synthesis aligned to the review objective. **Results:** Studies from 13 countries were included and classified as quantitative (*n* = 16; 51.6%), qualitative (*n* = 11; 35.5%), or mixed-methods (*n* = 4; 12.9%). A total of 34 instruments were used, but only one was specifically designed to evaluate eating attitudes and behaviors in children and adolescents with CD. **Conclusions:** Analysis of disordered eating in children and adolescents with CD remains methodologically heterogeneous and evolving. Quantitative studies predominate but often rely on non-specific instruments that blur the distinction between adaptive dietary vigilance and disordered eating. Qualitative and mixed-methods approaches highlight lived experiences and reveal measurement gaps despite their higher costs. Progress depends on developing specific instruments for this population that better capture the complexity of GFD management across development.

## 1. Introduction

Celiac disease (CD) is a chronic, systemic autoimmune enteropathy induced by gluten consumption in genetically predisposed individuals. A dysregulated immune response to gluten exposure results in inflammation and histological damage to the small intestinal mucosa, leading to atrophy of the intestinal villi and associated clinical manifestations. However, due to the systemic nature of CD, an increasing number of extraintestinal symptoms have been recognized as part of its clinical spectrum [1]. In terms of epidemiology, it is estimated that CD affects roughly 1% of the world’s population, with prevalence rates varying according to gender, age, and geographic location. In the pediatric population, a systematic review demonstrated that biopsy-confirmed prevalence is approximately twice that observed in adults, despite underreporting [2].

Despite the advancement of studies, the only effective treatment for CD currently remains the gluten-free diet (GFD), which requires the complete lifelong exclusion of all foods containing gluten—a protein fraction found in wheat, rye, and barley. Although essential for achieving clinical remission and preventing complications, the GFD is strict and restrictive for patients with CD, as it must eliminate widely consumed and culturally valued foods [3,4,5]. The strict adherence to a GFD can be particularly difficult for children and adolescents. Adherence rates in this population vary widely, ranging from 23% to 98%, with a median of 78%, and are influenced by individual, family, and contextual factors [6]. Furthermore, even among CD patients with good adherence to a GFD, the possibility of unintentional transgression, especially through cross-contamination, remains a recurring concern, impacting not only clinical outcomes but also emotional health and quality of life [7].

In this sense, after diagnosis, eating habits are guided by safety criteria that profoundly transform the way the individual relates to food. Beyond nutritional value or personal preferences, eating might become a practice mediated by planning, control, and risk anticipation [8,9,10]. Under these conditions, adherence to the GFD can assume a subjective complexity, influenced not only by the presence of clinical symptoms but also by cultural, emotional, and social factors. It is a process that may demand cognitive skills for decision-making, behavioral strategies for navigating diverse contexts, and communication skills for managing social situations involving food, especially outside the home, where safety and predictability are lower. In these contexts, concern about the availability of safe food and the constant need for vigilance can generate discomfort, a perception of difference, feelings of exclusion, and a reduced sense of belonging [11,12,13].

The analysis of these experiences related to eating can be deepened by examining eating attitudes and behaviors. Eating attitudes can be understood as beliefs, thoughts, feelings, and predispositions related to food consumption, representing the relationship with the act of eating [14]. Eating behavior, in turn, refers to eating practices, which involve how, when, where, and with whom one eats, shaped by a wide variety of sociocultural, biological, and psychological factors [15]. Identifying and understanding these multiple determinants is essential for translating scientific knowledge into healthier eating practices adapted to personal needs [16].

Although there is growing interest in understanding the complexity of the GFD in children and adolescents with CD, a diversity of methodological approaches for assessing these dimensions persists. Recent studies indicate that the instruments used in this field are, for the most part, adaptations of scales developed for other clinical conditions and aimed predominantly at adult populations, which may limit sensitivity to specific aspects of CD in children and adolescents, such as fear of cross-contamination, food hypervigilance, impact on autonomy, and social exclusion [11,12]. Furthermore, the literature highlights the lack of methodological standardization, and the absence of instruments developed through direct listening to children and adolescents [13].

The diversity of methods and lack of standardization suggest this field is still emerging, underscoring the need for greater conceptual clarity and stronger methodological foundations. Therefore, this scoping review aims to systematically map how disordered eating behaviors related to GFD management are assessed in children and adolescents with CD, describing the range of methodological approaches used, and identifying gaps in current research.

## 2. Materials and Methods

### 2.1. Study Design and Registration

This scoping review was conducted in line with established guidelines of the Joanna Briggs Institute (JBI) methodology for scoping reviews [17] and followed the reporting guidelines of the Preferred Reporting Items for Systematic Reviews and Meta-Analyses extension for Scoping Reviews (PRISMA-ScR) [18]. The following research question guided the review: “What methodological approaches are used to assess eating behaviors in children and adolescents with celiac disease?”. The process involved five key stages: defining the research question, identifying relevant studies, selecting eligible studies, mapping the extracted data, and analyzing and reporting the findings [17,18]. A registered protocol was lodged with the Open Science Framework (OSF) in January 2026 (https://doi.org/10.17605/OSF.IO/WYCES).

### 2.2. Eligibility Criteria

The eligibility criteria were developed using the mnemonic Patient, Concept, and Context (PCC) approach. In this framework, patient refers to children and adolescents (0–19 years, according to the World Health Organization [19]); the concept encompasses methodological approaches used to assess disordered eating behaviors; and the context concerns GFD management and its psychological, social, and cultural implications in any setting. Studies were excluded according to the following reasons: (1) Studies conducted exclusively with adults; (2) Studies involving mixed populations without age-specific stratification; (3) Studies that include other gluten-related conditions without clearly differentiating results specific to CD; (4) Studies focusing on parental strategies and attitudes rather than on children or adolescents with CD; (5) Studies that assess only nutritional, clinical, or GFD adherence outcomes; (6) Studies with insufficient conceptual clarity to support the identification of disordered eating attitudes and/or behaviors; (7) Studies that did not explore eating-related experiences associated with daily GFD management in children or adolescents with CD; and (8) Publication types: reviews, expert opinions, commentaries, letters to the editor, conference reports, blogs, and other non-peer-reviewed materials as well as studies exclusively focused on the translation and/or validation of instruments without application in a study sample.

### 2.3. Search Strategy

The search strategy followed three stages, as recommended by the JBI methodology for scoping reviews [17]: (i) an initial limited search in MEDLINE (via PubMed) and Scopus (Elsevier) to identify relevant terms by analyzing the keywords contained in the titles and abstracts, as well as the indexing terms to describe the retrieved studies; (ii) development and implementation of a comprehensive search strategy, which combines all terms identified in the previous step, which were adapted and applied to all select databases; and (iii) manual search of the reference list of the included studies to identify additional sources that may not have been captured in the previous searchers [17].

The search strategy was initially developed in PubMed, which served as the reference for adapting it to the other databases to ensure consistency in concepts and structure. In total, the search was conducted across ten databases: MEDLINE (via PubMed), Scopus (Elsevier), Embase (Elsevier), LILACS (via BVS), Web of Science (Clarivate), ProQuest Dissertation & Theses Global Citation Index (Clarivate), LIVIVO (ZB MED), PsycINFO (APA), Google Scholar, and TRIP Medical Database—no restrictions were applied to geography, language, or year of publication. The search terms used, Boolean combinations, truncation symbols, proximity operator, and database-specific filters are detailed in Supplementary Table S1. The last search was conducted on 19 January 2026.

### 2.4. Selection Process

Before the study selection process, all references identified across databases were imported into EndNote Online Classic (Thomson Reuters, Philadelphia, PA, USA) to organize and automatically remove duplicate entries. The resulting file was exported to Rayyan (Rayyan Systems Inc., Cambridge, MA, USA), where reviewer 1 [R1 (MCS)] manually verified and removed any remaining duplicates. The selection process was conducted in two phases (Phase 1 and Phase 2) and followed predefined eligibility criteria, with reviewers (R1) and [R2 (CRS)] blinded to each other’s decision throughout. Any discrepancies between R1 and R2 at either phase were discussed until consensus was reached. When consensus could not be reached, reviewer 3 [R3 (RD)] was consulted to make the final decision.

Phase 1 began with a calibration exercise involving 25 references, aimed at aligning the application of eligibility criteria and ensuring consistency in workflow between reviewers. Subsequently, titles and abstracts were independently screened using the Rayyan software (Rayyan Systems Inc., Cambridge, MA, USA). Phase 2 involved independent full-text screening of the studies selected in Phase 1, with support of a Microsoft Excel spreadsheet to document selection decisions. This phase was also preceded by a calibration exercise involving the full-text articles using five studies. Studies excluded in Phase 2 and the respective reasons for exclusion were summarized in Supplementary Table S2.

### 2.5. Data Extraction

To maintain methodological rigor, R1 and R2 independently extracted duplicate data while blinded to each other’s selections. Initially, a data extraction table was developed in Microsoft Excel and refined by the reviewers based on a deductive approach grounded in the PCC framework [20]. During table development, a preliminary data extraction was performed using a sample of 20 studies to assess its adequacy for addressing the research questions. Adjustments were made as needed throughout this process. The data items extraction table was organized into four main blocks: (i) study identification; (ii) population characteristics; (iii) study aim; and (iv) methodological approaches used and domains related to disordered eating attitudes and behaviors. Subsequently, R3 compared the extracted data to verify consistency and accuracy across reviewers, and any discrepancies were resolved through discussion until consensus was reached.

### 2.6. Data Analysis and Synthesis

The data analysis for this scoping review involved a descriptive mapping of methodological approaches used to assess disordered eating attitudes and behaviors in children and adolescents with CD. Data extraction utilized two complementary tables. Additionally, a figure was created to visually synthesize the distribution of included studies, for this purpose, Gemini (Google, Mountain View, CA, USA) was used to generate a Python script (Python Software Foundation, Beaverton, OR, USA). The analysis emphasized a descriptive examination of methodological characteristics within the evidence base. Studies were initially categorized by methodological approach (quantitative, qualitative, or mixed methods) and then grouped into analytical subcategories within each approach. To support organization and synthesis of the extracted data, the authors used NotebookLM (Google, Mountain View, CA, USA) and ChatGPT (OpenAI, San Francisco, CA, USA).

Results were organized by category to facilitate cross-study comparison and support a clear presentation of findings. In addition, a narrative synthesis was conducted to contextualize the mapped methodologies in relation to the review objective and research question. This synthesis provided a comprehensive overview of the methodological approaches used in the included studies, identified recurring patterns, highlighted underrepresented areas within the current evidence base, and outlined gaps relevant to future research.

## 3. Results and Discussion

**Figure 1:**
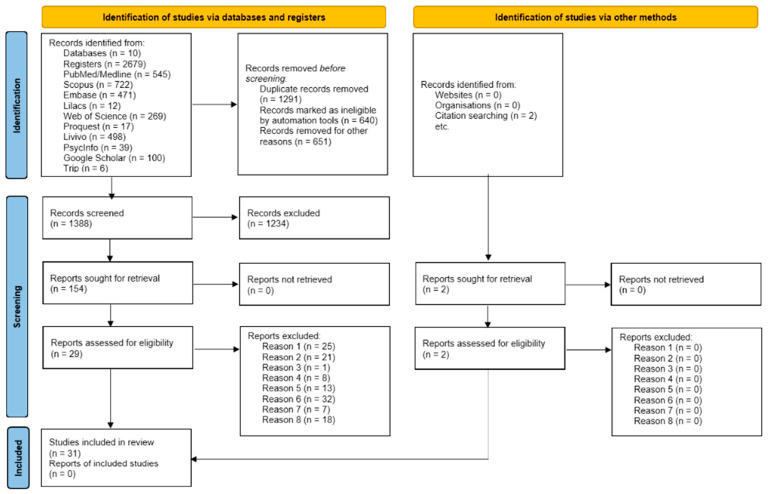
Flow diagram of literature search and selection criteria.

Once the systematic literature search and analysis were complete, a total of 2679 records were identified in the databases, of which 1291 were removed as duplicates. The remaining 1388 records were screened by title and abstract, and 154 articles were selected for full-text assessment. Additionally, reference list screening identified two eligible studies. Overall, 31 studies met the eligibility criteria and were included in the review. The entire selection process, including the number of records at each stage, was described in detail using a PRISMA flow diagram (Figure 1).

### 3.1. Study Characteristics

**Figure 2:**
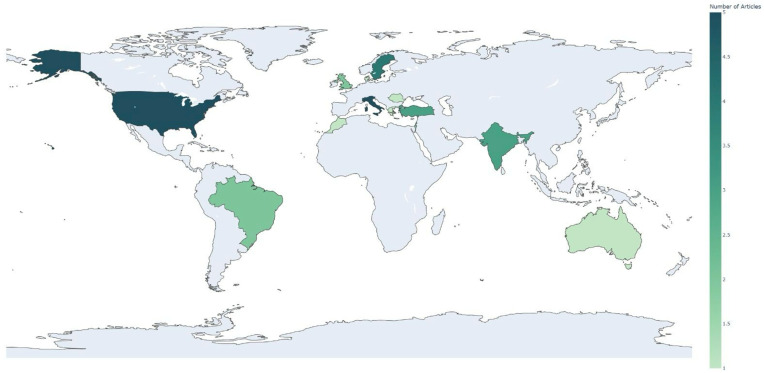
Worldwide distribution of included studies.

Studies on this topic have been produced in 13 countries (Figure 2), highlighting the geographical and cultural diversity of the research. The highest concentration of studies comes from Italy (*n* = 5) and the United States (*n* = 5), followed by Sweden (*n* = 4), India (*n* = 3), and Türkiye (*n* = 3). There were also contributions from Brazil (*n* = 2), Israel (*n* =2), and the United Kingdom (*n* = 2), while Australia, Denmark, Greece, Morocco, and Romania each contributed with only one study. This distribution highlights the global relevance of eating-related challenges in CD across diverse sociocultural contexts.

### 3.2. Characterization of Methodological Approaches

Data on methodological approaches used to assess eating behaviors in children and adolescents with CD can be divided into three main groups: qualitative, quantitative, and mixed-methods studies. Regarding study design, the data show a predominance of quantitative studies, representing 16 studies (51.6%). Qualitative research comes next, with 11 studies (35.5%), resulting in a ratio of approximately 1.4 quantitative studies per qualitative study (Table 1). In addition, a smaller group of 4 publications (12.9%) used mixed methods, combining both research approaches, trying to provide a more comprehensive examination of the topic.

### 3.3. Quantitative Studies

Quantitative research prioritizes the objective measurement of reality using numerical data and predetermined protocols to ensure measurement validity and allow broad generalization [51]. Consistent with this approach, most studies in this review employed quantitative methods to establish statistical correlations between clinical and behavioral variables and facilitate objective comparisons with healthy control groups. Reflecting the reliance on standardized apparatus, the review identified 34 standardized instruments used to evaluate disordered eating attitudes or behaviors (Table 2).

**Figure 3:**
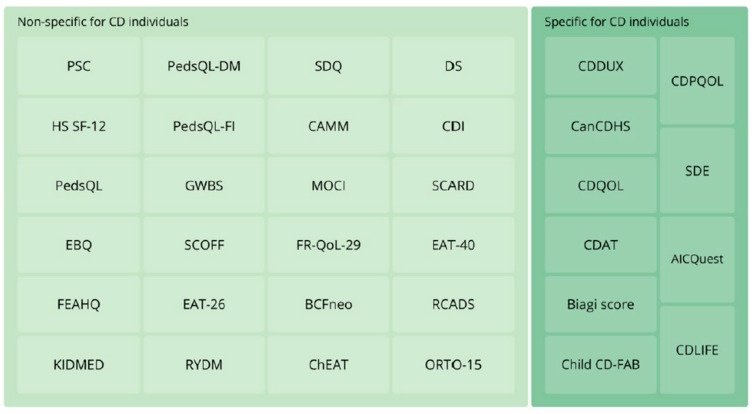
Quantitative instruments applied across the included studies (interactive Figure: https://www.canva.com/design/DAHAlbN9Wic/HXWxTI3uarm5_Cslv2Rh7g/view?utm_content=DAHAlbN9Wic&utm_campaign=designshare&utm_medium=link2&utm_source=uniquelinks&utlId=h47e321aeea).

These instruments were often employed in combination with other questionnaires or integrated with sociodemographic, clinical, and contextual data, resulting in composite methodological approaches rather than stand-alone measures. Only a subset (*n* = 10) was specific to CD, as represented in interactive Figure 3 [7,42,47,52,53,54,55,56,57,58]. However, this methodological reliance on standardization may impose significant limitations on the depth of its findings. By restricting the analysis to statistical data, quantitative research often provides only a general understanding. This approach tends to capture events at a single point in time, offering a static picture that may overlook the dynamic processes or specific contexts that may contribute to the observed results. Furthermore, excessive emphasis on objectivity can limit the interpretation of participants’ evaluation of their own behavior, ultimately sacrificing detailed understanding in favor of broad generalization. In contrast, the quantitative approach may stand out for its ability to provide a representative view of society, as the use of large, randomly selected samples enables statistical extrapolation of results to the general population or specific subsets. Additionally, this methodology is distinguished by its logistical efficiency, as the application of predetermined protocols and the use of analysis software reduce data collection and processing time, enabling objective analysis, which is often favored for defining new policies [51].

AICQuest: Associazione Italiana Celiachia Structured Questionnaire; BCFneo: Brazilian Children’s Food Neophobia Questionnaire (BCFNeo); CAMM: Child and Adolescent Mindfulness Measure; CanCDHS: Canadian Coeliac Health Survey; CDAT: CD Adherence Test; CDLIFE: CD Life Impact for Children; CDPQOL: Celiac disease Pediatric quality of life; CDQOL: CD-specific quality of life; CDDUX: CD-specific DUX questionnaire; CDI: Children’s Depression Inventory; ChEAT: Children’s Eating Attitude Test; Child CD FAB: Child CD Food Attitudes and Behaviours Scale; EAT-40: Eating Attitudes Test; EQB: Eating Behaviors Questionnaire; FEAHQ: Family Eating and Activity Habits Questionnaire; FR-QoL-29: Food-Related QOL questionnaire-29; GWBS: General Well-Being Scale; HS SF-12: Health Status Questionnaire Short Form-12; KIDMED: Mediterranean Diet Quality Index in Children and Adolescents; MOCI: Maudsley Obsessive–Compulsive Inventory; PedsQL: Pediatric QOL questionnaire Generic Core Scale; PedsQL-DM: Pediatric QOL questionnaire Diabetes Module (Version 3.2); PedsQL-FI: Pediatric QOL questionnaire Family Impact Scale (Module); PSC: Pediatric Symptom Checklist; ORTO-15: Questionnaire for the diagnosis of orthorexia; RCADS: Revised Child Anxiety and Depression Scale; RYDM: Resilience Youth Development Module; SCARD: Screen for Child Anxiety and Related Disorders; SCOFF: Sick Control Fat Food questionnaire; SDQ: Strengths and Difficulties Questionnaire.

The assessment of disordered eating attitudes and behaviors in CD faces a central methodological challenge, particularly in quantitative research. There is currently no consensus on the most suitable instruments to capture the complexities of a context in which strict, lifelong adherence to a GFD is the sole treatment option. Consequently, the literature has employed a diverse range of assessment instruments, reflecting both the complexity of the phenomenon, the evolution of the methodological strategies adopted and different analytical priorities, which can be grouped into: disease-specific experiences of living with CD [31,45]; eating pathology and maladaptive patterns [37,38,40,43,46,48]; broader psychosocial functioning screening [23,26,36,39]; quality-of-life assessments [11,24]; and dietary habits and adherence behavior [33,41].

In this context, CD-specific instruments have become increasingly important, as they enable the assessment of aspects of the GFD experience that generic measures do not adequately capture. The Celiac Disease Dutch Questionnaire (CDDUX) [52], a disease-specific health-related quality-of-life measure, is particularly notable in this regard. For example, in young individuals with comorbidities such as type 1 diabetes (T1D) and CD, quality of life, as measured by the Pediatric Quality of Life Inventory (PedsQL) [61], was similar across groups. However, the CDDUX identified lower scores specifically related to the emotional burden of adhering to a lifelong diet [31]. Furthermore, evidence from a multicenter cohort demonstrates that the CDDUX can distinguish the most sensitive domains of the CD experience, with the “Having Celiac Disease” domain identified as the most critical. This domain, which assesses feelings about the availability of gluten-containing foods and persistent concerns about eating, consistently received the lowest scores. These findings reveal a disease-specific burden that often remains undetected when only generic quality-of-life instruments, such as the PedsQL, are employed [45].

Behaviors associated with eating disorder risk have been investigated using non-specific screening instruments originally validated in the general population. Among the included studies, different versions of the Eating Attitudes Test (EAT) [69,79,80] were used, selected based on the study population and analytical focus, including applications in adolescents [37,40], research on orthorexia [48], and pediatric samples with comorbid T1D and CD [46]. Brief screening instruments, such as the SCOFF questionnaire [68], were also used to identify core symptoms of classical eating disorders [38]. Instruments designed to assess specific eating patterns have also been incorporated, including the ORTO-11 [82] (an adapted version of the ORTO-15 [83]) to assess orthorexic tendencies in adolescents with and without CD [48] as well as the Food Neophobia Scale [78] to quantify reluctance to try new foods [43].

Although these instruments enable comparisons with healthy populations [40,48] and with other chronic conditions [46], their application in patients with CD may be constrained by the overlap between treatment-related behaviors and symptoms often associated with eating disorders. This duality is evident in analyses of orthorexia and food neophobia, where strict control over ingredients [48] and reluctance to try new foods [40,43] often function as defensive mechanisms against accidental gluten ingestion. Similarly, high disgust sensitivity and fear of contamination have been associated with disordered eating attitudes, but may also reflect the hypervigilance necessary to maintain food safety [40]. The fear of gluten presence in food is a legitimate concern for CD individuals, driving the necessary vigilance to avoid symptoms and intestinal damage [84].

The distinction between the dietary vigilance necessary for the management of CD and disordered eating behaviors is central to understanding the experiences of children and adolescents with CD. While strict adherence to a GFD is the only effective treatment, how this adherence is experienced and implemented can vary from adaptive vigilance to maladaptive patterns that resemble eating disorders. Adaptive vigilance refers to the behaviors and attitudes necessary to maintain food safety and adherence to GFD without resulting in significant psychological distress or functional impairment. It is characterized by flexibility, confidence, and a non-obsessive awareness of cross-contamination risks, allowing for a balanced food-related quality of life [35,84,85].

In contrast, disordered eating arises when necessary vigilance becomes excessive and rigid, possibly resulting in psychosocial impairment and reduced quality of life, frequently characterized by rigidity, avoidance, controlling behavior, excessive worry, and social isolation. In general, adaptive vigilance is motivated by health maintenance and pragmatic safety, whereas disordered eating in CD patients is frequently driven by excessive fear of contamination and a need for absolute control, which might be common due to its potential health effects associated with frequent reports of inadvertent gluten consumption in food service establishments and in products with inadequate information and labeling [35,84,85]. Failure to distinguish adaptive vigilance from psychopathology may increase, possibly resulting in an overestimation of eating disorder prevalence. To address this potential source of misclassification, methodological refinements in the administration of assessment scales are required, such as instructing participants to disregard medically prescribed dietary restrictions when responding to items about weight-loss desire [37] and ensuring that therapeutic adherence is not misclassified as psychopathology.

Further, some authors have focused on psychosocial functioning and highlighted the need for specific instruments to supplement generic mental health scales, as standardized measures often miss the complexities of living with CD. To address this, structured interviews were developed alongside the Pediatric Symptom Checklist (PSC) [59] to assess social barriers and emotional responses to dietary management. These instruments revealed that dietary transgressions are mainly linked to anger about restrictions and shame in social situations [23,26], and that these feelings often persist even after clinical improvement and reduced overall stress [36]. More recently, a prospective study examined psychosocial functioning by exploring resilience and mindfulness in disease self-management [39]. Sequential assessments of mindfulness, resilience, psychosocial difficulties, and dietary adherence revealed a paradox: while dietary adherence improved, mindfulness and resilience declined, suggesting possible psychological resource depletion from sustained dietary control [39].

Beyond immediate psychosocial functioning, the methodological analysis of quality-of-life indicators suggests that the perceived impact of CD may vary across instruments and comparative populations. The combination of an ad hoc lifestyle questionnaire with the Questionnaire on the Health Status Short Form-12 [60] suggested preserved physical health and poorer mental health, associated with strategies of social avoidance and dietary restriction, such as avoiding restaurants and carrying personal food [24]. In contrast, to isolate the food-related experience, one study employed the Food-Related Quality of Life (FR-QoL-29) [77], developed initially for inflammatory bowel disease and later adapted for CD, allowing comparison between the two conditions [11]. Within this approach, eating behavior was characterized as a potential pattern of cognitive hypervigilance associated with ongoing diet management. Taken together, these contrasts show that the characterization of eating behaviors can be powerfully shaped by the measurement strategies adopted [11].

Another methodological approach to assessing eating behaviors involves analyzing large-scale trends through longitudinal comparative designs supported by population-based surveys. In this context, the Italian Celiac Association [42] developed a self-administered questionnaire on eating habits and distributed it via official digital channels, enabling comparison of results from two distinct time points (2011 and 2022). Over time, increased dietary adherence and decreased temptation to deviate from the diet were observed, alongside the adoption of self-protective strategies and increased social withdrawal [41].

Finally, the impact of the GFD on eating behaviors was further examined in the family context using the Family Eating and Activity Habits Questionnaire (FEAHQ) [66]. This approach triangulated data by having each family member complete the questionnaire, allowing for statistical comparisons within households. Results indicate that dietary restrictions for one family member can lead to changes in family eating practices, including increased consumption of ultra-processed foods and less structured eating patterns. The analysis also identified consistent differences between adolescents’ self-reports and parental proxy-reports, highlighting that the dietary burden varies by caregiving role and underscoring the importance of using multiple information sources to assess the psychosocial impact of treatment [33,39].

Across the included studies, an additional methodological distinction emerges regarding the source of information, ranging from a single subject responding to children and caregivers as sources. The simultaneous application of instruments to children/adolescents and caregivers was used to assess quality of life, psychopathological symptoms, and family habits [31,33,39,45,48], also including the combination of interviews with children and parental questionnaires [23]. In contrast, exclusive parental reports were more prevalent in psychosocial screenings or with younger children [26,36,43], while self-reporting was preferred in investigations of eating experiences and behaviors among adolescents [11,24,37,38,40,41,46].

This heterogeneity revealed systematic discrepancies, with parents reporting worse overall quality of life than their children [45]. Furthermore, complex associations, such as those between anxiety and eating attitudes, were detected only in self-report [48]. Even so, greater agreement was observed in directly observable behaviors, such as family habits and peer relationships [33,39], suggesting that using children and caregivers as informants allows for capturing different perspectives on the same phenomenon, while using a single informant limits the evidence to a single point of view.

### 3.4. Qualitative Studies

Qualitative research focuses on the linguistic understanding of social processes, prioritizing the depth of human meanings and motivations over statistics. It has a flexible and interactive design that allows constant adjustments to keep pace with reality as a subjective construct. In this framework, neutrality is replaced by the researcher’s role as both subject and object, making data analysis inseparable from interpretation [51].

In the included qualitative studies employing focus group interviews, data collection proceeded until theoretical saturation was achieved, ensuring comprehensive exploration of all relevant topics. In a study [28], a focus group discussion explored the impact of CD and GFD on health-related quality of life. The sessions were guided by a topic guide that enabled participants to direct the discussion according to their own concerns. The analysis was conducted using Grounded Theory [86], involving line-by-line reading and inductive coding of transcripts. This approach resulted in the identification of two major categories: “having CD” and “coping with CD”, reflecting key aspects of disease experience and coping [28].

In other studies that employed focus group interviews, research conducted by the same group [21,22], analyzed the same dataset using two approaches: an initial analysis based on Grounded Theory [86], and a subsequent reanalysis using a stigma-based theoretical framework [87] to further investigate social dimensions. The dataset consisted of discussions in which adolescents articulated their beliefs, perceptions, expectations, attitudes, needs, and experiences related to the chronic disease and its dietary management. The initial analysis identified what participants do in managing a GFD [21], whereas the subsequent analysis explored why social and contextual processes influence these behaviors, revealing themes such as “being a celiac”, “relationships”, and “eating a gluten-free diet”. Dietary adherence was characterized as a duality between concealing and disclosing the disease, a decision influenced by environmental and social group factors [22].

Regarding semi-structured interviews, there was more methodological diversity in data analysis. In one study, family histories were analyzed by combining Grounded Theory and narrative analysis, using specialized software to organize the data and focusing the analyses on social interactions [29]. In another study, the researchers adopted a phenomenological approach to understand participants’ experiences and the meanings they attribute to them, using an analysis process based on continuous identification of themes from the data and comparison of reports [44]. A third study sought to identify patterns of meaning through Reflexive Thematic Analysis, adopting a view of meanings as context-dependent and relating data analysis to previously existing theoretical frameworks [49].

When analyzing the semi-structured interviews used by the authors, despite methodological differences, common analytical dimensions emerge across the studies. The first dimension concerns illness identity, particularly regarding the construction of “being celiac”. Illness identity refers to the process by which a chronic condition is integrated into one’s sense of self and everyday life [88]. Qualitative accounts capture how diagnosis and food-related restrictions are gradually incorporated into daily routines and social interactions. A study used open-ended questions about the history of diagnosis and dietary management [29], while another explored this dimension of the disease by asking subjects what they think CD is [44]. A further study addressed identity by exploring children’s experiences at the moment of diagnosis and the situations they found challenging, emphasizing the child’s perspective [49].

A second shared dimension concerns perceptions of safety at home and risks encountered outside the home. All studies contrast the home, described as a safe and controlled environment, with external settings that introduce uncertainty and vulnerability. Family strategies for maintaining the GFD were explored [29], while experiences in educational institutions focused on relationships with friends and teachers in the school context [44], and the management of cross-contact inside and outside the home was also examined [49]. Across these studies, social isolation emerged as a deliberate coping strategy to ensure dietary control in the face of external risks [29,44,49].

Finally, the emotional responses associated with risk emerged as a third fundamental dimension. Anxiety, fear, and guilt were recurrent across the studies, although they manifested with specific nuances. On one hand, social participation and dependence on others, noting that fear of stigma or of being perceived as a burden often leads to social isolation [29]. On the other hand, guilt related to children’s perception of themselves as a financial or social burden results in shame and attempts to conceal the disease [44]. Additionally, fear of contamination can escalate into hypervigilance, characterized by excessive monitoring of the environment and imagined chains of contamination [49].

Taken together, the studies using semi-structured interviews indicate consistent patterns in eating-related behaviors across contexts. Dietary management and associated eating behaviors might change depending on the environment: the home is seen as a safe space, whereas external environments generate anxiety that requires compensatory strategies. Common strategies include avoiding food prepared by others, bringing lunch boxes, or simply not eating in situations of doubt, such as when traveling or at school. The studies clarify that eating is not just a nutritional practice but rather a complex behavior shaped by the trust that children place in their caregivers and peers [29,44,49].

Although they also employed semi-structured interviews, some studies differed in adopting Content Analysis as their analytical approach [30,32,50]. Whereas the previously discussed studies emphasized identity and the phenomenology of illness, these authors used Content Analysis [89,90,91] to structure the data around specific analytical perspectives, such as comorbidity between T1D and CD [30], gender norms related to femininity [32] and the feasibility of clinical interventions, including the Gluten-Free Resilience and Overall Wellness (GROW) project [50], offering a more pragmatic and segmented view of the challenges involved in managing a GFD.

In this context, a study explored the experiences of children and adolescents living with T1D and CD. They used the open-ended question “Tell me what it is like to live with two diseases,” to examine the challenges associated with comorbid T1D and CD, emphasizing the social restrictions imposed by a GFD [30]. A further study explored the everyday life experiences of young women diagnosed with CD through a screening program. Narratives were analyzed from a gender perspective, demonstrating that norms of femininity and concerns about “not being a burden” impede dietary adherence [32]. Another study with adolescents and their caregivers applied this approach to organize feedback from both groups on a clinical intervention (the GROW Project), identifying needs related to resilience and the development of communication skills [50]. Overall, these studies extend the previous discussion by shifting attention from the subjective experience of “being celiac” to the identification of contextual and demographic barriers influencing treatment management [30,32,50].

Finally, in contrast to focus groups or semi-structured interviews, qualitative content analysis applied to written narratives allowed the exploration of experiences in a more private context, without the direct mediation of an interviewer. From this perspective, the Critical Incident Technique [92] was applied in a study involving children and adolescents diagnosed with CD to identify dilemmas associated with the GFD across different contexts, showing that the feeling of being different arises not only from dietary restrictions but also from forced adaptive behaviors, such as bringing one’s own meals or declining social invitations [25]. Similarly, a study of adolescents, based on a five-year longitudinal follow-up after diagnosis via population screening, identified the internalization of perceived risk as a central element of the experience, demonstrating that, in the absence of prior symptoms, adherence to a GFD results from a gradual process of adaptation to daily routines, supported by growing understanding and acceptance of future risks [27].

Despite its explanatory potential regarding the why and how of social phenomena, which stems from its interpretive depth and flexible design, qualitative research faces significant methodological limitations, including high interpretive demands, prolonged analysis time, and a reliance on small samples. These factors restrict the generalizability of findings and hinder their use in contexts that demand speed and statistical representativeness, potentially leading to a preference for quantitative approaches in decision-making processes and public policy formulation [51].

### 3.5. Mixed-Methods Studies

Mixed methods research is characterized by the integration of quantitative and qualitative data to optimize the strengths and counterbalance the weaknesses of each independent approach. From an applied perspective, this method combines complementary approaches to address complex problems, connecting contextual understanding with quantifiable results. By rigorously integrating data, the mixed-methods approach enables a more comprehensive understanding of phenomena, encompassing both their dimensions and the meanings people attribute to them [93]. This method has been used to deepen understanding of eating attitudes and behaviors associated with GFD management, notably by expanding and contextualizing findings from quantitative assessments.

Across included studies, qualitative approaches have been incorporated both to refine the interpretation of quantitative results [34,35] and to support the development of measures sensitive to children’s and adolescents’ experiences [7,47]. A study of adolescents and adults analyzed how quality of life is associated with energy levels, adherence, and knowledge of GFD [34]. Another study of adolescents aimed to understand dietary management strategies and their impact on quality of life [35]. However, the complex effects of strict adherence in young people are not always captured by standardized measures, highlighting the lack of specific assessment instruments for this population [35]. To access these nuances, both studies adopted mixed-methods designs, combining quantitative assessment, such as the Standardized Dietitian Evaluation (SDE) [56] and CD-specific quality-of-life measures, with qualitative approaches. The qualitative component relied on semi-structured interviews to explore the lived experience of dietary management, guided by open-ended questions. Whereas the first one focused on perceived barriers and facilitators to maintaining a strict GFD [34], the other focused on identifying adaptive and maladaptive behavioral patterns [35].

Taken together, the findings indicate that dietary management does not operate as a simple dichotomy between adherence and nonadherence, but rather as a set of distinct behavioral patterns associated with distinct quality-of-life outcomes. In both studies, greater dietary adherence was associated with poorer quality of life [34,35]. In a study conducted with adults and adolescents, high dietary adherence, assessed by the SDE, was directly interpreted as indicative of an extreme vigilance pattern, based on the degree of dietary control observed. However, this association raises important conceptual concerns: in the context of CD, strict adherence to a GFD is a fundamental clinical requirement [34]. Interpreting high adherence as inherently problematic, therefore, potentially carries the risk of pathologizing behaviors that are largely necessary and expected, particularly when variability in motivations, strategies, and meanings attributed to dietary control is not considered.

Finally, as mentioned previously, mixed-methods designs were fundamental to the development of assessment of quantitative instruments sensitive to the experiences of children and adolescents with CD, thereby helping to fill methodological gaps already identified in the literature. In this context, the Coeliac Disease Food Attitudes and Behaviours Scale (CD-FAB) [94] was adapted for the pediatric population [7], acknowledging the lack of instruments specifically validated for this age group [7]. For this, the author conducted cognitive interviews using the think-aloud technique [95], allowing children aged 8 to 13 years to evaluate and reformulate items related to cross-contamination and trust in others. These qualitative contributions informed the final version of the scale, which was subsequently subjected to psychometric analyses to assess its internal consistency and validity, resulting in the first instrument specifically designed to assess eating attitudes and behaviors in children and adolescents with CD: the Child CD-FAB [7].

Similarly, a mixed-methods approach was used to develop the Celiac Disease Life Inventory of Family Experiences (CDLIFE), a disease-specific quality-of-life questionnaire developed through a three-stage process. The development began with concept elicitation interviews with children, adolescents, and parents to generate items grounded in real-life experiences; this was followed by cognitive refinement interviews, culminating in psychometric testing to validate the instrument. Although designed as a quality-of-life measure, the final version integrates specific factorial domains—Adaptive Vigilance and Eating Behaviors/Adjustment—that enable the quantitative assessment of how food-related vigilance and eating-related social dynamics associated with the GFD shape well-being in this population [47].

Despite the numerous advantages and strengths of the design, this methodological depth poses significant challenges in execution. Because it involves multiple data collection and analysis methods, mixed-methods research frequently requires more time and budget than single-method studies. Furthermore, the merging point between the two single methods presents analytical difficulties, especially when quantitative and qualitative findings appear contradictory. Therefore, successfully applying this approach generally requires multidisciplinary teamwork to address the theoretical and technical differences between the methods, making the process resource-intensive and requiring advanced methodological knowledge [94].

## 4. Conclusions

This scoping review demonstrates that the literature presents a methodologically and conceptually heterogeneous field that is still consolidating. A central challenge identified in the reviewed studies is the interpretation of eating-related behavior associated with GFD management. The finding indicates that a comprehensive understanding of these behaviors in children and adolescents with CD might require a more nuanced, contextually informed perspective of their relationship with food, particularly regarding the therapeutic demands of a GFD, the degree of distress associated with these behaviors, and their potential impact on biopsychosocial well-being. This conceptual challenge is also reflected in the methodological approaches used across the included studies.

Quantitative studies predominate, using instruments originally developed for other populations and clinical conditions, which limits the ability to capture central aspects of the food experience in children and adolescents with CD. While these instruments may allow population comparisons and statistical analyses, many do not adequately distinguish adaptive behaviors necessary for adherence to a GFD, such as food vigilance and risk avoidance, from potentially dysfunctional manifestations, which raises the possibility of overestimation of disordered eating behaviors. In this context, qualitative studies might deepen understanding of the meanings attributed to food, highlighting recurring dimensions such as identity, food security, fear of contamination, social stigma, and coping strategies, although they may have limitations related to the generalizability of findings and the costs and time required for data collection and analysis. By integrating these perspectives, mixed-methods studies emerge as a potentially valuable strategy, integrating objective measurement with an understanding of lived experience, possibly contributing to both the critical interpretation of quantitative results and the development of more sensitive and specific instruments.

The review identifies a clear gap in the availability of validated instruments specifically for children and adolescents with CD, which is partially addressed by instruments recently developed from direct listening to this population. Thus, it is concluded that advancing the field depends on strengthening conceptual and methodological foundations, with greater investment in approaches that differentiate therapeutic adaptation to a GFD, incorporate multiple informants, and prioritize specific instruments for children and adolescents with CD. These efforts are essential to produce more precise, clinically useful, and ethically sensitive evidence regarding the complexity of managing a GFD throughout development.

## Figures and Tables

**Table 1:** Overview of included studies assessing eating attitudes and behaviors in children and adolescents with celiac disease.

Author, Year	Reference	Objective	Study Design	Population	Methodological Approaches
Olsson et al., 2008	[21]	To explore how adolescents with CD perceive and manage their everyday lives in relation to a GFD.	Observational, cross-sectional study with a qualitative approach	Adolescents with CD (*n* = 47); 68.08% female; aged 15–18 years	Focus group interviews; data collection guided by a topic guide with open-ended questions; qualitative analysis based on Grounded Theory (open coding, categorization, and theme development)
Olsson et al., 2009	[22,23]	To report adolescent experiences in circumstances in which their medical condition becomes visible to others if they choose or request only the gluten-free foods appropriate to their condition, and how they manage the consequences.	Observational, cross-sectional study with a qualitative approach	Adolescents with CD (*n* = 47); 68.08% female; aged 15–18 years	Focus group interviews; data collection guided by a topic guide with open-ended questions; qualitative reanalysis of focus group transcripts [21] using a stigma-based theoretical framework
Chauhan et al., 2010	[23]	To assess dietary compliance with the GFD, to identify barriers to compliance and to study the impact of diet on the psychosocial behavior of children with CD.	Observational, cross-sectional study with a quantitative approach	Children and Adolescents with CD (*n* = 64); aged 2–17 years	Dietary assessment (detailed dietary history and clinical evaluation by a senior consultant); interview with a self-administered structured questionnaire covering demographic, clinical, and diet-related psychosocial factors; and the Pediatric Symptom Checklist
Altobelli et al., 2013	[24]	To assess HRQOL in children and adolescents with CD and explore how several demographic and clinical characteristics and GFD adherence affect their perceived health status.	Observational, cross-sectional study with a quantitative approach	Children and Adolescents with CD (*n* = 140); 78,6% female; mean age (SD) 14.2 (2.5)	Structured questionnaire adapted from the Canadian Coeliac Health Surve; Questionnaire on the Health Status SF-12 (Italian version); and CD-specific self-report survey including demographic, clinical, and diet-related QOL data, complemented by clinician validation
Biagetti; Naspi; Catassi, 2013	[25]	To evaluate the emotional impact of GFD on the everyday life of children affected by CD.	Observational, cross-sectional study with a qualitative approach	Children and Adolescents with CD (*n* = 76); 76,31% female; mean age (SD) 8.7	Critical Incident Technique-based qualitative study using written open-ended reports of diet-related dilemmas and qualitative categorization of lived experiences
Garg; Gupta, 2014	[26]	To identify the predictors of compliance with GFD in children with CD.	Observational, cross-sectional study with a quantitative approach	Children and Adolescents with CD (*n* = 134): Compliant group (*n* = 88), 69.8% female; mean age 6 years; Noncompliant group (*n* = 46), 30.2% female, mean age 8 years	Dietary compliance based on a 5-day dietary recall form; self-administered questionnaire on demographic, clinical, dietary compliance, and diet-related psychosocial factors; and Pediatric Symptom Checklist
Nordyke et al., 2014	[27]	To describe adolescents’ experience living with screening-detected CD five years after diagnosis to explore how their perceptions, practices, and beliefs evolved.	Observational, cross-sectional study with a qualitative approach	Adolescents (*n* = 153), 53.59% female, mean age one year follow-up 14.6; mean age five-year follow-up 18.0	Written narratives; qualitative content analysis with coding, categorization, and theme development
Skjerning et al., 2014	[28]	To explore the impact of CD and GFD on HRQOL in the everyday living of children and adolescents.	Observational, cross-sectional study with a qualitative approach	Children and adolescents (*n* = 23; 69.6% female; aged 8–18 years Parents (*n* = 3)	Focus group interviews; data collection guided by a topic guide supported by visual food stimuli; and qualitative analysis based on Grounded Theory
Bacigalupe; Plocha, 2015	[29]	To examine the barriers that families with a celiac child face and the strategies they use to adhere to the recommended diet.	Observational, cross-sectional study with a qualitative approach	Families (*n* = 10); children aged 6–12 years	Semi-structured family interviews with open-ended questions; qualitative analysis using grounded theory and narrative analysis
Brancaglioni et al., 2016	[30]	To understand the experience of children and adolescents living with T1D and CD.	Observational, cross-sectional study with a qualitative approach	Children and Adolescents (*n* = 5), 60% female, aged 10–16 years	Semi-structured interviews; family and social context mapping using genogram and ecomap; qualitative content analysis conducted within a symbolic interactionism framework
Pham-Short et al., 2016	[31,32]	To evaluate the QOL and glycemic control in youth with T1D and CD vs. T1D only. We hypothesized that QOL scores would be lower in youth with T1D and CD and in those who were nonadherent to the GFD.	Observational, cross-sectional study with a quantitative approach	Youth with T1D (*n* = 35), 54% female, mean age (SD) 13.6 (3.0); Youth with T1D and CD (*n* = 35), 57% female, mean age (SD) 13.7 (3.1)	Demographic and clinical data collection; Pediatric QOL Generic Core Scale (version 4.0); Pediatric QOL Inventory Diabetes Module (version 3.2); Pediatric QOL Inventory Family Impact Scale; General Well-Being Scale; modified Eating Behaviors Questionnaire; CD-specific DUX questionnaire; and GFD adherence assessed clinically and serologically
Kautto et al., 2017	[32]	To study the experiences of the everyday life among Swedish young female patients who were diagnosed with CD through a screening study.	Observational, cross-sectional study with a qualitative approach	Young females with CD (*n* = 7); 100% female; aged 17–18 years	Semi-structured individual interviews; qualitative content analysis with inductive coding from manifest to latent themes and gender-informed interpretation
Levran et al., 2018	[33]	To assess the influence of GFD on the child and his/her family’s eating habits and lifestyle behaviors.	Observational, prospective study with a quantitative approach	Children with CD (*n* = 40); 52.2% female; mean age (SD) 7.4 (2.8)	Demographic and anthropometric data collection; assessment of family obesogenic environment using the Family Eating and Activity Habits Questionnaire; symptoms and GFD compliance questionnaire
Wolf et al., 2018	[34]	To examine the associations between QOL and energy levels and adherence to, and knowledge about, a GFD.	Observational, prospective study with a quantitative and qualitative approach	Teenagers (*n* = 30); 80% female; mean age (SD) 15.7 (1.5) Adults (*n* = 50); 84% female; mean age (SD) 23.3 (3.4)	Demographic and medical history data collection; CD-specific QOL instruments (CD QOL Questionnaire; CD Pediatric QOL Questionnaire); dietary adherence assessed by the Standardized Dietitian Evaluation instrument and one item from the CD Adherence Test; knowledge assessed by a food label quiz; and open-ended questions on barriers and facilitators to GFD adherence analyzed using thematic analysis
Cadenhead et al., 2019	[35]	To understand adolescents’ approaches to managing a GFD and the association with QOL	Observational, prospective study with a quantitative and qualitative approach	Adolescents with CD (*n* = 30); 80% female; mean (SD) age 15.6 (1.5) years	Demographic and medical history data collection; CD Pediatric QOL questionnaire; Celiac Dietary Adherence Test; and semi-structured interviews exploring barriers and facilitators to GFD adherence, analyzed using a psychosocial rubric-guided qualitative approach
Chellan et al., 2019	[36]	To study QOL in pediatric CD and the effect of a GFD in a North Indian population.	Observational, prospective study with a quantitative approach	Children with CD (*n* = 44); 31.8% female; mean age (SD) 6.03 (0.42)	Baseline demographic/clinical/biochemical assessment; Pediatric Symptom Checklist; and 6-month post-GFD disease-specific structured questionnaire adapted from Chauhan et al., assessing adherence, barriers, parental behaviors and perceptions, and children’s feelings
Tokatly Latzer et al., 2020	[37]	To assess the incidence and risk factors for disordered eating behaviors (DEB) among individuals with CD, and to examine the association between adherence to GFD and DEBs.	Observational, cross-sectional study with a quantitative approach	Adolescents with CD (*n* = 136); 63% female; mean age (SD) 13.3 (1.2)	Web-based survey collecting demographic and clinical data; Eating Attitudes Test-26; and GFD adherence questionnaire (Biagi score)
Zriouel; Cherkani-Hassani; Khadmaoui, 2020	[38]	To evaluate dietary habits, to screen for eating disorders, and to identify the factors associated with eating disorders among adolescents with CD.	Observational, cross-sectional study with a quantitative approach	Children and adolescents with CD (*n* = 132); 59.84% female; aged 10–16 years	Demographic and clinical data collection; Mediterranean diet quality assessment using the Mediterranean Food Quality Index for Children and Adolescents; eating disorders screening using the Sick Control Fat Food questionnaire; and anthropometric assessment (body mass index)
Maddison-Roberts, 2023	[7]	To gain a comprehensive understanding of how children with CD experience and navigate the GFD, focusing on their dietary preferences, perceptions, and challenges, as well as the impact of these experiences.	Observational, cross-sectional study with a qualitative approach	Children and Adolescents (*n* = 15); 60% female; mean age 10.5	Semi-structured online interviews guided by a theoretical model of eating behaviors in CD and family consultation; qualitative analysis based on reflexive thematic analysis
Bozas et al., 2024	[39]	To investigate the impact of psychological factors on behavioral and dietary responses in adolescents with CD, utilizing repeated measures over time.	Observational, prospective study with a quantitative approach	Children and adolescents (*n* = 31), 61.3% female, mean age (SD) 13.6 (2.06); Parents (*n* = 27)	Baseline demographic and disease-related medical history and the Resilience Youth Development Module; longitudinal follow-up including the Celiac Dietary Adherence Test, Strengths, and Difficulties Questionnaire—child version, and Child and Adolescent Mindfulness Measure; and post-follow-up resilience reassessment and parent-reported Strengths and Difficulties Questionnaire
Efe; Tok, 2024	[40]	To investigate the impact of obsessive–compulsive symptomatology and disgust propensity on disordered eating attitudes (DEA) and poor GFD compliance in adolescents with CD.	Observational, cross-sectional study with a quantitative approach	Celiac group (*n* = 148), 58,4% female, mean age (SD) 14.6 (0.33); Control group (*n* = 104), 71.1% female, mean age (SD) 14.63 (0.36)	Demographic, socioeconomic, family-related, and clinical data; Eating Attitudes Test-26; Maudsley Obsessive–Compulsive Inventory; Disgust Scale adapted; assessment of GFD adherence using serological markers and self-report; Children’s Depression Inventory; Screen for Child Anxiety and Related Disorders
Fiori et al., 2024	[41]	To describe Italian celiac patients who agreed to participate in the latest web survey and their attitudes toward the GFD (compliance, perceived limitations, and worries) and to compare the answers given by the 2011 and 2022 responders.	Observational, cross-sectional study with a quantitative approach	Children and adolescents (*n* = 477): Group I: (*n* = 173), 69% female, aged ≤10 years; Group II: (*n* = 163), 63% female, aged 11–14 years; Group III: (*n* = 141), 61% female, aged 15–17 years	Self-administered structured questionnaire developed by the Associazione Italiana Celiachia, described in Corposanto et al. [42], comprising multiple-choice items, Likert-scale statements, and open-ended questions, with a subset of 50 items 50 used in the present study
Matran et al., 2024	[11]	To evaluate comparative food-related QOL in light of these dietary interventions between these two conditions.	Observational, cross-sectional study with a quantitative approach	Children with chronic gastrointestinal disorders (*n* = 51): CD (*n* = 17), 58.8% female, median age (IQR) 13 (10.8–16.4); Crohn’s disease (*n* = 17), 47.1% female, median age (IQR) 16 (12–16.75); Ulcerative colitis (*n* = 17), 70.6% female, median age (IQR) 16 (14–117).	Demographic and clinical data; history of surgery and medical treatment regimens; adherence to the GFD (for patients with CD); anthropometric measures (weight, height) and body mass index; self-reported Food-Related QOL questionnaire, semantically adapted for CD.
Nogueira Firme et al., 2024	[43]	To investigate and classify the prevalence of food neophobia among Brazilian children aged 4 to 11 years with gluten-related disorders.	Observational, cross-sectional study with a quantitative approach	Children with Gluten-Related Disorders (*n* = 209); 57.9% female; mean age (SD) 7.2 (2.3)	Sociodemographic, clinical data, and Food Neophobia questionnaire (caregiver-reported)
Sahın et al., 2024	[44]	To examine in depth the disease management experiences of school- age children with CD and the effects of family, friends, and teachers on disease management at school.	Observational, cross-sectional study with a qualitative approach	Children and adolescents (*n* = 14); 71.4% female; mean age 12	Semi-structured interviews; phenomenological qualitative approach with grounded theory-informed analysis
Crocco et al., 2025	[45]	To assess HRQOL and further characterize the clinical factors associated with reduced HRQOL, in a large multicenter pediatric cohort with CD.	Observational, cross-sectional study with a quantitative approach	Children and adolescents (*n* = 857); 66.4% female; mean age (SD) 12.9 (2.9)	Clinical and sociodemographic data collection; The disease-specific questionnaire CD Dutch Questionnaire; and Generic questionnaire Pediatric QOL Inventory
Franceschi et al., 2025	[46]	To analyze the prevalence of disordered eating behaviors in T1D + CD and T1D individuals, and to identify the main predictors.	Observational, cross-sectional study with a quantitative approach	Children and Adolescents with T1D and CD (*n* = 66), 51% female, mean age (SD) 13.8 (2.6); Children and adolescents with T1D only (*n* = 84), 39.3% female, mean age (SD) 13.3 (1.6)	Children’s Eating Attitude Test; Body Mass Index z-score; diabetes-related data; CD-related data; dietary interview conducted by expert dietitians.
Coburn et al., 2025	[47]	To develop a CD-specific pediatric QOL measure (CD Life Inventory of Family Experiences) with parallel self-report and parent-report forms by generating items through concept elicitation interviews, iterative refinement using cognitive debriefing interviews and evaluating its psychometric properties and validity.	Observational, cross-sectional study with a quantitative and qualitative approach	Phase 1 (Concept Elicitation): 9 youth and 10 parents. Phase 2 (Item Refinement): 3 youth, 3 parents, and 8 clinicians. Phase 3 (Validation): Youth with CD (*n* = 102), 61.8% female, mean age (SD) 12.6 (3.0); Parent-proxy report (*n* = 103), 55.3% female, mean age (SD) 10.29 (3.74)]	Semi-structured concept elicitation interviews and qualitative content analysis for development of the CD Life Impact for Children; cognitive debriefing interviews for item refinement; and psychometric testing using the Patient-Reported Outcomes Measurement Information System, the Pediatric QOL Inventory and Family Impact Module, and the GFD–Visual Analog Scale, with self-report and parent-proxy versions
Gercek et al., 2025	[48]	To compare orthorexia nervosa symptoms, eating attitudes, anxiety, depression, and obsessive–compulsive disorder symptoms between adolescents with and without CD in this study.	Observational, cross-sectional study with a quantitative approach	Adolescents CD (*n* = 30); 73.3% female; mean age (SD) 15.93 (1.14) Healthy controls (*n* = 30); 63.3% female; mean age (SD) 16.13 (1.00)	Sociodemographic data collection; GFD adherence; Eating Attitudes Test; Revised Child Anxiety and Depression Scale—child and parent versions; and questionnaire for the diagnosis of orthorexia-11 scale (short version of questionnaire for the diagnosis of orthorexia-15)
Maddison-Roberts; Jones; Satherley, 2025	[49]	To increase understanding of children and adolescents’ approaches to GFD management and to improve identification of those with hypervigilant approaches. Part A: to improve understanding of interactions and experiences with food in children and adolescents with CD and explore how these relate to models of gluten-related distress and unhelpful eating developed in adults. Part B: to develop and validate a children and adolescent version of the CD–Food Attitudes and Behaviours to support the identification of children and adolescents with hypervigilant approaches to managing the GFD. The study also aims to improve understanding of the relationship between hypervigilance to the GFD in children and adolescents, CD-related health outcomes, and QOL.	Part A: Observational, cross-sectional study with a qualitative approach Part B: Observational, cross-sectional study with a quantitative approach	Part A: children and adolescents with CD (*n* = 15); 60% female; aged 8–13 years Part B: (a) Children and adolescents with CD (*n* = 15), 60% female, aged 8–13 years; (b) Children and adolescents with CD (*n* = 84), 61% female, mean age (SD) 11.35 (2.49)	Mixed-methods instrument development study using semi-structured online interviews and think-aloud cognitive interviewing analyzed through reflexive thematic and content analysis, followed by psychometric validation of the Child CD–Food Attitudes and Behaviours Scale, using the CD–Food Attitudes and Behaviours Scale, Pediatric QOL Inventory, State-Trait Anxiety Inventory for Children, Illness Perception Questionnaire–Revised, and Food Neophobia Test Tool.
Vagadori et al., 2025	[50]	To develop and refine the Gluten-Free Resilience and Overall Wellness Project, the first family-centered, online behavioral intervention to improve QOL and GFD self-management in adolescents with CD and their parents.	Observational, prospective study with a qualitative approach	Adolescents with CD and their caregivers: Round 1: (*n* = 5), 80% female, mean age (SD) 14.2 (1.1); Round 2: (*n* = 4), 75% female, mean age (SD) 13.3 (0.5)	Semi-structured stakeholder interviews, conducted in two iterative rounds, to adapt and refine a group-based telehealth behavioral intervention (Gluten-Free Resilience and Overall Wellness Project); following an inductive approach to qualitative analysis

Abbreviations: CD—celiac disease; GFD—gluten-free diet; HRQOL—health-related quality of life; SD—standard deviation; QOL quality of life; T1D—type 1 diabetes.

**Table 2:** Characteristics and structure of instruments used in the included studies.

Instrument	Reference of the Studies Included that Used the Instrument	Target Population	Respondent	Structure and Scoring	Items and Domains	Specific to Evaluate Eating Attitudes/Behaviors
**Specific for CD individuals**						
Focus group interview	[21,22,28]	Children and adolescents with CD	Self-report	Open-ended questions; qualitative thematic analysis; no scoring system	N/A	Yes
CD-specific DUX questionnaire [52]	[31,45]	Children and adolescents with CD aged 8–18 years	Self-report and parents-proxy	5-point Likert scale (smiley answer categories); transformed to 0–100 scale	12-item; 3 subscales: Diet (6 items), Communication (3 items), and Having CD (3items)	No
Canadian Coeliac Health Survey [53]	[24]	Children with CD aged <16 years	Parent-proxy	Mixed response formats; descriptive epidemiological survey; no standardized subscales or total score	76-item; multiple thematic sections	No
Written narratives	[27]	Adolescents with screening-detected CD, aged 12–18 years	Self-report	Written narrative prompts; qualitative content analysis; no scoring system	No predefined items or domains; studies explore main thematic areas emerging from qualitative analysis.	Yes
Semi-structured interviews	[29,30,32,44,49,50]	Children, adolescents, and young adults with CD	Self-report	Open-ended questions; qualitative thematic or content analysis; no scoring system	No predefined items or domains; studies explore main thematic areas emerging from qualitative analysis.	Yes
CD-Specific QOL [54]	[34]	Adults and adolescents with CD	Self-report	5-point Likert scale (“not at all” to “a great deal”); score range 0–100 points	20-item; 4 clinically relevant subscales: Dysphoria (4 items), Limitations (9 items), Health Concerns (5 items), and Inadequate Treatment (2 items)	No
CD Pediatric QOL [55]	[34,35]	Children and adolescents with CD aged 8–18 years	Self-report	5-point Likert scale (“never” to “almost always”) (0–4); score range 0–100 scale	Two age-specific versions: 8–12 years (13 items; subscales: negative emotions, school, enjoyment) and 13–18 years (17 items; subscales: social, uncertainty, isolation, and limitations.	No
Standardized Dietitian Evaluation [56]	[34]	Adolescents and adults with CD	Dietitian (Interview/Dietary Recall)	Interview and three 24 h dietary recalls; 6-point Likert scale; ranging 1 (excellent adherence) to 6 (not currently following a GFD)	N/A	No
CD Adherence Test [56]	[34,35,39]	Adults with CD	Self-report	5-point Likert scale; score range 7–35 points	7-item; unidimensional construct	No
Biagi score [57,58]	[37]	Patients with CD (primarily adults)	Self-report	Score range 0–4	4-item; unidimensional construct	No
Child CD Food Attitudes and Behaviors Scale [7]	[7]	Children and adolescents aged 8–16	Children and adolescents (self-report)	7-point Likert scale (1 “strongly agree” to 7 “strongly disagree”); Score ranges 14–98 points	14 items; Unidimensional	Yes
Associazione Italiana Celiachia Structured Questionnaire [42]	[41]	Elderly, adults, adolescents and children with CD, aged 10 and over	Self-report	Semi-structured type questionnaire; mostly closed-ended questions with some open-ended items; no standardized scoring system	50-item; five sections: personal details (master data), information about associationism, quality of life, eating habits and transgressions food;	No
CD Life Impact for Children [47]	[47]	Children and adolescents with CD	Self-report and parents- proxy	5-point Likert scale; score range 1–5 (mean of items)	21-item; 4 subscales: social impact, external support and functioning, adaptative vigilance and eating behaviors and adjustment; the proxy version includes a single item on financial resources	No
**Non-specific for CD individuals**						
Pediatric Symptom Checklist [59]	[23,26,36]	Children and adolescents aged 4–15 years	Parent-proxy	3-point frequency scale (never, sometimes, and often); score range 0–75	35-item; unidimensional construct	No
Health Status Questionnaire Short Form-12 [60]	[24]	General population and clinical adult populations	Self-report	Mixed, item-specific categorical response formats; two summary scores (physical health section and mental health section) based on weighted item combinations.	12-item; 8 health concepts: physical functioning, role limitation-physical, bodily pain, general health, role limitation-emotional, vitality, mental health, social functioning	No
Pediatric QOL questionnaire Generic Core Scale (version 4.0) [61]	[31,45,47]	Children and adolescents aged 2–18 years	Self-report and parents-proxy	5-point Likert scale; transformed to 0–100 scale	23-item; 4 subscales: physical functioning (8 items), emotional functioning (5 items), social functioning (5 items), and school functioning (5 items)	No
Pediatric QOL questionnaire Diabetes Module (Version 3.2) [62]	[31]	Children, Adolescents, and Young Adults with T1D ages 2–25 years	Self-report and parents-proxy	5-point Likert-type scale; transformed to a 0–100 scale	33-item; 5 subdomains: diabetes symptoms (15 items), treatment barriers (5 items), treatment adherence (6 items), worry (3 items), and communication (4 items)	No
Pediatric QOL questionnaire Family Impact Scale (Module) [63]	[31,47]	Parents of children with complex chronic health conditions	Self-report	5-point response scale scored (“never a problem” to “always a problem”); transformed to a 0–100 scale	36-item; 8 Subdomains: physical functioning (6 items), emotional functioning (5 items), social functioning (4 items), cognitive functioning (5 items), communication (3 items), worry (5 items), daily activities (3 items), and family relationships (5 items)	No
General Well-Being Scale [64]	[31]	Adults aged 25–74 years	Self-report	6-point ordinal scale (0–5); total score and subscale scores	18-item; 6 subscales: anxiety (3 items), depressed mood (3 items), positive well-being (4 items), self-control (3 items), general health (3 items), vitality (2 items)	No
Eating Behaviors Questionnaire [31] [adapted from [65]]	[31]	Children with T1D, with or without CD	Self-report and parents-proxy	5-option response format (“never” to “almost always”); score range 0–100	23-item; subdomains: individual child eating behaviors, family and social environment eating behavior, and diabetes-related eating behaviors	Yes
Family Eating and Activity Habits Questionnaire [66]	[33]	Families with children aged 6–12 years	Self-report	Scores calculated for each member of the family	32-item; 4 separate scales: leisure time activity (items 1–4), eating habits and style (items 5–16), response to internal hunger and satiety cues (items 17–19), stimulus exposure and control (items 20–32)	Yes
Mediterranean Diet Quality Index in Children and Adolescents [67]	[38]	Children and adolescents aged 2–24 years	Self-report and proxy-report	Dichotomous (Yes/No); score range 0–12	16-question; unidimensional construct	No
Sick Control Fat Food questionnaire [68]	[38]	Adults	Self-report	Dichotomous (Yes/No); score range 0–5	5-question; unidimensional construct	Yes
Eating Attitudes Test-26 [69]	[37,40]	Adolescents and Young Women	Self-report	6-point Likert scale (“never” to “always”) (0–3); score range 0–78 points	26-item; 3 Subscales: dieting, bulimia and food preoccupation and oral control	Yes
Resilience Youth Development Module [70]	[39]	Adolescents aged 11–18 years	Self-report	4-point Likert scale, (“not at all true” to “very much true”)	51-item; 12 assets (8 Environmental assets and 4 Internal Assets)	No
Strength and Difficulties Questionnaire [71]	[39]	Parents and children aged 3–16 years	Self-report and proxy-report	3-point Likert scale (“not true”, “somewhat true”, or “certainly true”)	25-item; 5 scales (5 items each): hyperactivity/inattention, emotional symptoms, conduct problems, peer problems, prosocial behavior	No
Child and Adolescent Mindfulness Measure [72]	[39]	Children and adolescents aged 10–17	Self-report	5-point Likert scale (“never true” to “always true”); score range 0–40 points	10-item; unidimensional construct	No
Maudsley Obsessive–Compulsive Inventory [73]	[40]	Adults	Self-report	Dichotomous scale (True/False); total score and subscale scores	30-item; 4 subscales: checking, cleaning, slowness, doubt	No
Children’s Depression Inventory [74,75]	[40]	Children and adolescents	Self-report	3-point rating scale (0–2); score range 0–54	27-items; unidimensional score	No
Screen for Child Anxiety and Related Disorders [76]	[40]	Children and adolescents	Self-report and parent-proxy	3-point rating scale (0–2); total score and factor scores	38-item; 5 factors: somatic/panic, general anxiety, separation anxiety, social phobia, and school phobia.	No
Food-Related QOL questionnaire-29 [77]	[11]	Patients with inflammatory bowel disease aged ≥16	Patients (self-report)	5-point Likert response scale (“definitively agree” to “definitively disagree”; score range 29–145 points	29-item; Unidimensional	Yes
Food Neophobia questionnaire [78]	[43]	Caregivers of children aged 4–11 years, with gluten-related disorders	Caregivers of children (self-report)	5-point Likert response scale (response options: “totally disagree” to “totally agree”; “not at all” to “extremely”; “certainly not” to “certainly”; “never” to “always”); scores calculated as the sum of item values, both as total score and domain-specific scores	25-item three domains: neophobia in general; neophobia for fruits; neophobia for vegetables.	Yes
Children’s Eating Attitude Test [79]	[46]	Children aged 8–13 years	Self-report	6-point Likert scale (“never” to “always”); score range 0–130 points	26-items; 3 subscales: dieting, bulimia/food preoccupation, and oral control	Yes
Eating Attitudes Test [80]	[48]	Adolescents and adults’ women	Participants (self-report)	6-point Likert-type scale (“always” to “never”) (0–3); score range 0–78	40-item; 7 factors reflecting: food preoccupation, body image for thinness, vomiting and laxative abuse, dieting, slow eating, clandestine eating, and perceived social pressure to gain weight	Yes
Revised Child Anxiety and Depression Scale [81]	[48]	Child and parent forms	self-report scale	four-point Likert scale	47-item; 6 domains: Social Anxiety Disorder; Separation Anxiety; Generalized Anxiety Disorder; Panic Disorder; Obsessive–Compulsive Disorder Major Depressive Disorder	No.
Questionnaire for the diagnosis of orthorexia-11 scale [82] [short version of questionnaire for the diagnosis of orthorexia-15 [83]	[48]	Participants aged >16	Participants (self-report)	4-point Likert scale (0–3); scores range 15–60	15-item; 3 underlying factors: cognitive–rational (items 1, 5, 6, 11, 12, 14), clinical (items 3, 7, 8, 9, 15), and emotional (items 2, 4, 10, 13)	Yes

Abbreviations: CD—celiac disease; GFD—gluten-free diet; QOL—quality of life, T1D—type 1 diabetes.

## Data Availability

None.

## Supplementary Materials

The following supporting information can be downloaded at: https://www.mdpi.com/article/10.3390/nu18111661/s1, Table S1: Search Strategy; Table S2: Excluded articles and reasons for exclusion.

## Abbreviations

The following abbreviations are used in this manuscript:

BMI	Body mass index
CD	Celiac disease
CDDUX	Celiac Disease Dutch Questionnaire
CD-FAB	Coeliac Disease Food Attitudes and Behaviours Scale
CDLIFE	Celiac Disease Life Inventory of Family Experiences
DEB	Disordered Eating Behaviors
EAT	Eating Attitudes Test
FEAHQ	Family Eating and Activity Habits Questionnaire
GFD	Gluten-free diet
HRQOL	Health-related quality of life
JBI	Joana Briggs Institute
OSF	Open Science Framework
PedsQL	Pediatric Quality of Life Inventory
PRISMA-ScR	Preferred Reporting Items for Systematic Reviews and Meta-Analyses extension for Scoping Reviews
PCC	Patient, Concept, and Context
PSC	Pediatric Symptom Checklist
QOL	Quality of life
SD	Standard deviation
T1D	Type 1 diabetes
