# Supplementary material for: Methodological Approaches to Assess Disordered Eating Behaviors Related to Gluten-Free Diet Management in Children and Adolescents with Celiac Disease: A Scoping Review
Source: Nutrients. 2026 May 22;18(11):1661. doi: 10.3390/nu18111661 (PMC13258312; doi:10.3390/nu18111661)
Supplement: Supplementary file 1 [file nutrients-18-01661-s001.zip › nutrients-4289746-supplementary.pdf]

Table S1. Search strategy

Database (n=10)	Search strategy on January 19th, 2026
PubMed/MEDLINE	#1 "Celiac Disease"[Mesh] OR "celiac disease"[tiab] OR "coeliac disease"[tiab] OR "sprue celiac" OR "gluten enteropathy"[tiab:~3] OR "gluten enteropathies"[tiab:~3]<br>#2 "Feeding Behavior"[Mesh:NoExp] OR "Feeding and Eating Disorders"[Mesh] OR "Dietary Patterns"[Mesh] OR "Health Knowledge, Attitudes, Practice"[Mesh] OR behavior*[tiab] OR behaviour*[tiab] OR "eating attitudes"[tiab] OR "food attitudes"[tiab] OR "eating disorder"[tiab] OR "disordered eating"[tiab:~5] OR "eating pathology"[tiab] OR "food avoidance"[tiab] OR hypervigilance[tiab] OR "maladaptive eating"[tiab] OR "diet* habit*"[tiab] OR eating[tiab] OR "food related"[tiab:~5]<br>#3 "Diet, Gluten-Free"[Mesh] OR "Quality of Life"[Mesh] OR "Psychological Well-Being"[Mesh] OR "gluten free diet"[tiab] OR "life quality"[tiab:~5] OR "well being"[tiab] OR stigma[tiab] OR management[tiab] OR social[tiab] OR psychosocial[tiab] OR vulnerability[tiab] OR experience*[tiab] OR challenges[tiab]<br>#4 #1 AND #2 AND #3
Scopus	(TITLE-ABS-KEY ("celiac disease" OR "coeliac disease" OR "sprue celiac" OR (gluten W/3 enteropath*)) AND TITLE-ABS-KEY ("Feeding Behavior" OR "Eating behavior" OR "eating behaviour" OR "Dietary Patterns" OR "eating attitudes" OR "food attitudes" OR (disorder* W/5 eating) OR "food avoidance" OR hypervigilance OR "maladaptive eating" OR "dietary habits" OR (food W/5 related) OR eating) AND TITLE-ABS-KEY ("Gluten-Free Diet" OR Psychological OR "gluten free diet" OR (life W/5 QUALITY) OR "well being" OR stigma OR management OR psychosocial OR vulnerability OR experience* OR challenges))
Embase	#1 'celiac disease'/exp OR 'celiac disease' OR 'celiac disease':ti,ab,kw OR 'coeliac disease':ti,ab,kw OR 'sprue celiac' OR (('gluten' NEAR/4 'enteropathy'):ti,ab,kw) OR (('gluten' NEAR/4 'enteropathies'):ti,ab,kw)<br>#2 'feeding behavior'/de OR 'eating disorder'/exp OR 'dietary pattern'/exp OR 'attitude to health'/exp OR 'eating behavior':ti,ab,kw OR 'eating behaviour':ti,ab,kw OR 'eating attitudes':ti,ab,kw OR 'food attitudes':ti,ab,kw OR 'eating disorder':ti,ab,kw OR (('disordered' NEAR/5 'eating'):ti,ab,kw) OR 'food avoidance':ti,ab,kw OR 'hypervigilance':ti,ab,kw OR 'maladaptive eating':ti,ab,kw OR 'dietary habits':ti,ab,kw OR 'food-related':ti,ab,kw<br>#3 'gluten free diet'/exp OR 'quality of life'/exp OR 'psychological well-being'/exp OR 'gluten free diet':ti,ab,kw OR (('life' NEAR/5 'quality'):ti,ab,kw) OR 'well being':ti,ab,kw OR

	'stigma':ti,ab,kw OR 'management':ti,ab,kw OR 'social':ti,ab,kw OR 'psychosocial':ti,ab,kw OR 'vulnerability':ti,ab,kw OR 'experiences':ti,ab,kw OR 'challenges':ti,ab,kw<br>#4 #1 AND #2 AND #3<br>#5 #4 AND [embase]/lim
Lilacs	(("Doença celíaca" OR "Enfermidade celiaca" OR "Enteropatia Glúten Induzida" OR "Espru Celíaco" OR celiaquia OR esteарreia) AND ("Comportamento alimentar" OR "Conducta Alimentaria" OR "Comportamento relacionado à alimentação" OR "Conduta na Alimentação" OR "Hábito Alimentar" OR "Hábitos Alimentares" OR "Hábitos Dietéticos" OR "Padrão Alimentar" OR "Padrões Alimentares" OR "Tradições Alimentares" OR "Transtornos da Alimentação" OR "Trastornos de Alimentación" OR "Padrões dietéticos" OR "Padrones dietéticos" OR "Padrões de Alimentação" OR "Padrão de Alimentação" OR "Patrones de Alimentación" OR "Patron dietético" OR "Conhecimentos em Saúde" OR "Conocimientos em Salud" OR "Atitudes e Prática em Saúde" OR "Atitudes e Práticas em Saúde" OR "Transtorno Alimentar" OR "trastorno alimentario") AND ("Dieta Livre de Glúten" OR "Dieta Sin Glúten" OR "Qualidade de Vida" OR "Calidad de Vida" OR "Bem-Estar Psicológico" OR "Bienestar Psicológico" OR "Bem-Estar Psíquico" OR "Integridade Psicológica" OR "Mal-Estar Psicológico")) AND instance:"lilacsplus"
Web of Science	"celiac disease" OR "coeliac disease" OR "sprue celiac" OR (gluten NEAR/3 enteropath*) (Topic) and "Feeding Behavior" OR "Eating behavior" OR "eating behaviour" OR "Dietary Patterns" OR "eating attitudes" OR "food attitudes" OR (disorder* NEAR/5 eating) OR "food avoidance" OR hypervigilance OR "maladaptive eating" OR "dietary habits" OR (food NEAR/5 related) (Topic) and "Gluten-Free Diet" OR "Psychological Well-Being" OR "gluten free diet" OR (life NEAR/5 quality) OR "well being" OR stigma OR management OR social OR psychosocial OR vulnerability OR experience* OR challenges (Topic)
ProQuest™ Dissertations & Theses Citation Index	"celiac disease" OR "coeliac disease" OR "sprue celiac" OR (gluten NEAR/3 enteropath*) (Topic) and "Feeding Behavior" OR "Eating behavior" OR "eating behaviour" OR "Dietary Patterns" OR "eating attitudes" OR "food attitudes" OR (disorder* NEAR/5 eating) OR "food avoidance" OR hypervigilance OR "maladaptive eating" OR "dietary habits" OR (food NEAR/5 related) (Topic) and "Gluten-Free Diet" OR "Psychological Well-Being" OR "gluten free diet" OR (life NEAR/5 quality) OR "well being" OR stigma OR management OR social OR psychosocial OR vulnerability OR experience* OR challenges (Topic)
Livivo	(("celiac disease" OR "coeliac disease" OR "sprue celiac" OR "gluten enteropathy" OR "gluten enteropathies") AND ("Feeding Behavior" OR "Feeding and Eating Disorders" OR "Dietary Patterns" OR "Health Knowledge" OR behavior OR behaviour

	OR attitudes OR "eating disorder" OR "disordered eating" OR "food avoidance" OR hypervigilance OR "maladaptive eating" OR "dietary habit") AND (“Gluten-Free Diet" OR "Quality of Life" OR "Psychological Well-Being" OR "gluten free diet" OR "well being" OR stigma OR management OR social OR psycho-social))
PsycInfo	Keywords: "celiac disease" OR Keywords: "coeliac disease" OR Keywords: "sprue celiac" OR (Keywords: gluten adj3 entero-path*) AND Keywords: "Feeding Behavior" OR "Feeding and Eating Disorders" OR "Dietary Patterns" OR "Health Knowledge" OR behav* OR attitudes OR "eating disorder" OR (disordered adj5 eating) OR "food avoidance" OR hypervigi-lance OR "maladaptive eating" OR "diet* habit*" OR "food-re-lated" AND Keywords: "Gluten-Free Diet" OR Keywords: "Psy-chological Well-Being" OR Keywords: "gluten free diet" OR (Keywords: life adj5 quality) OR Keywords: "well being" OR Keywords: stigma OR Keywords: management OR Keywords: social OR Keywords: psychosocial OR Keywords: vulnerability OR Keywords: experience* OR Keywords: challenges
Google Scholar	(("celiac disease" OR "coeliac disease" OR "sprue celiac" OR "gluten enteropathy" OR "gluten enteropathies") AND ("Feed-ing Behavior" OR "Feeding and Eating Disorders" OR "Dietary Patterns" OR "Health Knowledge" OR behavior OR behaviour OR attitudes OR "eating disorder" OR "disordered eating" OR "food avoidance" OR hypervigilance OR "maladaptive eating" OR "dietary habit" OR food-related) AND (“Gluten-Free Diet" OR "Quality of Life" OR "Psychological Well-Being" OR "gluten free diet" OR "well being" OR stigma OR management OR so-cial OR psychosocial OR vulnerability OR experiences OR chal-lenges))
Trip Medical Database	"celiac disease" OR "coeliac disease" OR "sprue celiac" OR "glu-ten enteropathy" OR "gluten enteropathies", "Feeding Behavior" OR "Feeding AND Eating Disorders" OR "Dietary Patterns" OR "Health Knowledge" OR behavior OR behaviour OR attitudes OR "eating disorder" OR "disordered eating" OR "food avoid-ance" OR hypervigilance OR "maladaptive eating" OR "dietary habit" OR "food-related", "Gluten-Free Diet" OR "Quality of Life" OR "Psychological Well-Being" OR "gluten free diet" OR "well being" OR stigma OR management OR social OR psycho-social OR vulnerability OR experiences OR challenges

Table S2. Excluded articles and reasons for exclusion

Author, year	Exclusion reason
Abed et al., 2025 [1]	1
Al-Raee et al., 2012 [2]	6
Alhuzaim et al., 2024 [3]	2
Alkhayyat et al., 2021 [4]	2
Ansaldi et al., 1992 [5]	5
Anson et al., 1990 [6]	4
Araújo et al., 2012 [7]	2
Araújo et al., 2011 [8]	2
Araya et al., 2020 [9]	1
Arigo et al., 2012 [10]	1
Atkins et al., 2024 [11]	8
Atsawarungruangkit et al., 2020 [12]	2
Ayyıldız et al., 2024 [13]	6
Bellini et al., 2011 [14]	6
Bombaet al., 2015 [15]	8
Bozas et al., 2024 [16]	7
Bravo et al., 2011 [17]	6
Butwicka et al., 2017 [18]	5
Calsbeek et al., 2002 [19]	6
Cederborg et al., 2012 [20]	4
Cheung et al., 2025 [21]	5
Coppell et al., 2019 [22]	6
Crocco et al., 2024 [23]	5
Czaja-Bulsa et al., 2018 [24]	6
Daldaban Sarıca et al., 2025 [25]	6
Daneshdoost et al., 2023 [26]	8
Duerksen et al., 2016 [27]	8
Fernández Miaja et al., 2021 [28]	5
Fernández Miaja et al., 2021 [29]	5
Fishman et al., 2018 [30]	6
Franco et al., 2023 [31]	1
Fueyo-Díaz et al., 2018 [32]	6
Fueyo-Díaz et al., 2018 [33]	6
Fueyo-Díaz et al., 2020 [34]	1
Fueyo-Díaz et al., 2020 [35]	1
Gaeta et al., 2023 [36]	8
Garnweidner-Holme et al., 2020 [37]	1
Giannakopoulos et al., 2020 [38]	6
Grenet et al., 1975 [39]	5
Grootenhuis et al., 2007 [40]	6
Halmos et al., 2018 [41]	2
Hameed et al., 2023 [42]	4
Hoteit et al., 2022 [43]	2
Joelson et al., 2018 [44]	2
Kakkar et al., 2021 [45]	5
Kaltsa et al., 2015 [46]	3
Kalyoncu et al., 2017 [47]	8
Karwautz et al., 2008 [48]	2
Khurana et al., 2015 [49]	6
Kinos et al., 2012 [50]	6
Kinston et al., 1988 [51]	7

Kujawowicz et al., 2022 [52]	1
Kurppa et al., 2012 [53]	5
Lecerfet al., 2022 [54]	8
Leffler et al., 2007 [55]	1
Lu et al., 2018 [56]	6
Maddison-Roberts et al., 2024 [57]	8
Nimel et al., 2016 [58]	7
Mårildet al., 2017 [59]	5
Marsilio et al., 2020 [60]	1
Mazzone et al., 2011 [61]	6
Meyer et al., 2022 [62]	6
Meyer et al., 2021 [63]	6
Meyer et al., 2023 [64]	7
Meyer et al., 2016[65]	6
Meyer et al., 2017 [66]	6
Meyer et al., 2017 [67]	8
Meyer et al., 2018 [68]	6
Meyer et al., 2021 [69]	6
Meyer et al., 2017 [70]	8
Myléus et al., 2014 [71]	5
Nayar et al., 2009 [72]	6
Elesbão et al., 2024 [73]	1
Nunes et al., 2021 [74]	1
Okur-Ataş et al., 2025 [75]	6
Pancheva et al., 2025 [76]	1
Pozziani et al., 2011 [77]	8
Pratesi et al., 2018 [78]	1
Pynnönen et al., 2004 [79]	8
Pynnönen et al., 2005 [80]	7
Quick et al., 2012 [81]	1
Quick et al., 2011 [82]	1
Raber ,et al., 2023 [83]	8
Real Delor et al., 2024 [84]	2
Real-Delor et al., 2021 [85]	2
Riehl et al., 2022 [86]	8
Rimárová et al., 2018 [87]	6
Roma et al., 2010 [88]	6
Roos et al., 2013 [89]	1
Russo et al., 2020 [90]	4
Sady et al., 2025 [91]	6
Sainsbury et al., 2018 [92]	2
Sainsbury et al., 2013 [93]	1
Sainsbury et al., 2015 [94]	2
Satherley et al., 2017 [95]	1
Satherley et al., 2016 [96]	1
Satherley et al., 2017 [97]	1
Schilling et al., 2018[98]	5
Schwantes et al., 2025 [99]	8
Shah et al., 2014[100]	1
Shani et al., 2022 [101]	2
Silva et al., 2024 [102]	4
Silva et al., 2024 [103]	4
Silvester et al., 2016 [104]	2

Subramanian et al., 2024 [105]	7
Terrone et al., 2013 [106]	6
Tittel et al., 2021 [107]	7
Tokatly Latzer et al., 2018 [108]	2
Esenyel et al., 2014 [109]	6
Veen et al., 2013 [110]	4
Veen et al., 2010 [111]	2
Wagner et al., 2015 [112]	2
Wagner et al., 2008 [113]	2
Wagner et al., 2016 [114]	2
Wahab et al., 2019 [115]	6
Walsh et al., 2022 [116]	8
Weber et al., 2021 [117]	1
Weisbrod et al., 2025 [118]	6
Welch et al., 2015 [119]	5
Wolf et al., 2017 [120]	8
Wolf et al., 2018 [121]	8
Xhakollari et al., 2021 [122]	1
Zakharovaet al., 2021 [123]	4
Zingone et al., 2018 [124]	2
Zysket al., 2018 [125]	1

Exclusion reason for exclusion: 1. Studies conducted exclusively with adults; 2. Studies involving mixed populations without age-specific stratification; 3. Studies that include other gluten-related conditions without clearly differentiating results specific to celiac disease; 4. Studies focusing on parental strategies and attitudes rather than on children or adolescents with celiac disease; 5. Studies that assess only nutritional, clinical, or gluten-free diet adherence outcomes; 6. Studies with insufficient conceptual clarity to support the identification of disordered eating attitudes and/or behaviors; 7. Studies lacking a lived-experience perspective on eating in children or adolescents with celiac disease; 8. Publication types: reviews, expert opinions, commentaries, letters to the editor, conference reports, blogs, and other non–peer-reviewed materials, as well as studies exclusively focused on the translation and/or validation of instruments without application in a study sample.

1. Abed, M.T.; Abdo, Q.; Jaber, M.M.; Qatanani, A.A.; Raba'a, A.O.; Rabba, G.F.; Deeb, S.W.; Jallad, S.; Badrasawi, M. Eating Behaviors and Mental Health among Celiac Patients, Case-Control Study. *Indian J. Gastroenterol.* **2025**, *44*, 363–370, doi:10.1007/s12664-024-01732-w.
2. Al-Raee, M.B.; El-Sakka, M.A.; Al-Wahaidi, A.A. In Depth Analysis of Risk Factors for Coeliac Disease amongst Children under 18 Years Old in the Gaza Strip. A Cross Sectional Study. **2012**, doi:10.1186/1475-2891-11-97.
3. Alhuzaim, W.M.; AlDawas, O.D.; Alazmi, M.; AlMutairi, H.; Altoom, F.; AlShabanat, F.; Sabbah, B.N. Knowledge and Attitude of Celiac Disease Among the Population of Riyadh, Saudi Arabia. *Cureus* **2024**, *16*, doi:10.7759/cureus.68603.
4. Alkhayyat, M.; Qapaja, T.; Aggarwal, M.; Almomani, A.; Abureesh, M.; Al-otoom, O.; Zmaili, M.; Mansoor, E.; Abou Saleh, M. Epidemiology and Risk of Psychiatric Disorders among Patients with Celiac Disease: A Population-Based National Study. *J. Gastroenterol. Hepatol.* **2021**, *36*, 2165–2170, doi:10.1111/jgh.15437.
5. Ansaldi, N.; Dell'Olio, D.; Tavassoli, K.; Faussone, D.; La Vecchia, A.; Bramante, L. Aderenza Alla Dieta Ed Aspetti Sociali Dei Pazienti Con Malattia Celiaca. *Minerva Med* **1992**, *38*, 439–443.
6. Anson, O.; Weizman, Z.; Zeevi, N. Celiac Disease: Parental Knowledge and Attitudes of Dietary Compliance. *Pediatrics* **1990**, *85*, 98–103, doi:10.1542/peds.85.1.98.
7. Araújo, H.M.C.; Araújo, W.M.C. Coeliac Disease: Eating Habits and Quality of Life. *British Food Journal* **2012**, *114*, 1297–1309, doi:10.1108/00070701211258835.

8. Araújo, H.M.C.; Araújo, W.M.C. Coeliac Disease. Following the Diet and Eating Habits of Participating Individuals in the Federal District, Brazil. *Appetite* **2011**, *57*, 105–109, doi:10.1016/j.appet.2011.04.007.
9. Araya, M.; Bascuñán, K.A.; Alarcón-Sajarópulos, D.; Cabrera-Chávez, F.; Oyarzún, A.; Fernández, A.; Ontiveros, N. Living with Gluten and Other Food Intolerances: Self-Reported Diagnoses and Management. *Nutrients 2020, Vol. 12, Page 1892* **2020**, *12*, 1892, doi:10.3390/nu12061892.
10. Arigo, D.; Anskis, A.M.; Smyth, J.M. Psychiatric Comorbidities in Women with Celiac Disease. *Chronic Illn.* **2012**, *8*, 45–55, doi:10.1177/1742395311417639.
11. Atkins, M.; Michael, B.; Savage, M.; Kuo, B.; Murray, H.B.; Leonard, M.M. Su1327 PREVALENCE OF AVOIDANT/RESTRICTVE FOOD INTAKE DISORDER IN PEDIATRIC AND ADULT PATIENTS PRESENTING FOR CELIAC DISEASE CONSULTATION. *Gastroenterology* **2024**, *166*, S-730, doi:10.1016/s0016-5085(24)02156-5.
12. Atsawarungruangkit, A.; Silvester, J.A.; Weiten, D.; Green, K.L.; Wilkey, K.E.; Rigaux, L.N.; Bernstein, C.N.; Graff, L.A.; Walker, J.R.; Duerksen, D.R. Development of the Dietitian Integrated Evaluation Tool for Gluten-Free Diets (DIET-GFD). *Nutrition* **2020**, *78*, doi:10.1016/j.nut.2020.110819.
13. Ayyıldız, D.; Demirtaş, Z.; Kınacı, E. Psychiatric Difficulties in Children with Celiac Disease and the Relationship between Adherence to Treatment and Parental Attitudes. *Turk. J. Gastroenterol.* **2024**, *35*, 743–749, doi:10.5152/tjg.2024.23493.
14. Bellini, A.; Zanchi, C.; Martelossi, S.; Di Leo, G.; Not, T.; Ventura, A. Compliance with the Gluten-Free Diet: The Role of Locus of Control in Celiac Disease. *J. Pediatr.* **2011**, *158*, doi:10.1016/j.jpeds.2010.08.034.
15. Bomba, M.; Tremolizzo, L.; Corbetta, F.; Conti, E.; Riva, A.; Nacinovich, R. Anorexia Nervosa in Adolescent Girls and Celiac Disease Serology. *Eur. Child Adolesc. Psychiatry* **2017**, *24*, S132–S132, doi:10.1007/s00787-015-0714-4.
16. Bozas, A.; Karakatsoulis, G.; Panagopoulou, E.; Xinias, I.; Fotoulaki, M. Charting the Path: Psychological Factors and Diet Adherence in Adolescents With Celiac Disease. *Cureus* **2024**, *16*, doi:10.7759/cureus.74103.
17. Francisca Bravo, M.; María Paz Muñoz, F. Adherencia e Impacto de La Dieta Sin Gluten En Niños Con Enfermedad Celíaca. *Rev. Chil. Pediatr.* **2011**, *82*, 191–197, doi:10.4067/S0370-41062011000300003.
18. Butwicka, A.; Lichtenstein, P.; Frisén, L.; Almqvist, C.; Larsson, H.; Ludvigsson, J.F. Celiac Disease Is Associated with Childhood Psychiatric Disorders: A Population-Based Study. *J. Pediatr.* **2017**, *184*, 87-93.e1, doi:10.1016/j.jpeds.2017.01.043.
19. Calsbeek, H.; Rijken, M.; Bekkers, M.J.T.M.; Kerssens, J.J.; Dekker, J.; Van Berge Henegouwen, G.P.; Hopman, W.; Jansen, J.B.M.J.; Severijnen, R.S.V.M.; Tolboom, J.J.M.; et al. Social Position of Adolescents with Chronic Digestive Disorders. *Eur. J. Gastroenterol. Hepatol.* **2002**, *14*, 543–549, doi:10.1097/00042737-200205000-00012.
20. Cederborg, A.C.; Hultman, E.; Magnusson, K.F. Living with Children Who Have Coeliac Disease: A Parental Perspective. *Child Care Health Dev.* **2012**, *38*, 484–489, doi:10.1111/j.1365-2214.2011.01273.x.
21. Cheung, T.; McDonald, C.; Setty, M.; Tsai, P.; Wadhwani, S.I. Social Adversities Associate with Worse Disease Control in Pediatric Celiac Disease. *Journal of Pediatrics* **2025**, *276*, doi:10.1016/j.jpeds.2024.114305.
22. Copell, K.J.; Stamm, R.A.; Sharp, K.P.H. Diagnostic Delays and Treatment Challenges in Children with Coeliac Disease: The New Zealand Coeliac Health Survey Available online: https://www.researchgate.net/profile/Kirsten-Coppell/publication/337112305_Diagnostic_delays_and_treatment_challenges_in_children_with_coeliac_disease_The_New_Zealand_Coeliac_Health_Survey/links/5f7636d5299bf1b53e070c39/Diagnostic-delays-and-treatment-challenges-in-children-with-coeliac-disease-The-New-Zealand-Coeliac-Health-Survey.pdf (accessed on 9 February 2026).
23. Crocco, M.; Malerba, F.; Calvi, A.; Zampatti, N.; Gandullia, P.; Madeo, A.; Tappino, B.; Proietti, S.; Bonassi, S. Health-Related Quality of Life in Children with Coeliac Disease and in Their Families: A Long-Term Follow-up Study. *J. Pediatr. Gastroenterol. Nutr.* **2024**, *78*, 105–112, doi:10.1002/jpn3.12049.
24. Czaja-Bulsa, G.; Bulsa, M. Adherence to Gluten-Free Diet in Children with Celiac Disease. *Nutrients 2018, Vol. 10, Page 1424* **2018**, *10*, 1424, doi:10.3390/nu10101424.

25. Daldaban Sarıca, B.; Demirci, E.; Altay, D.; Arslan, D. Body Image Dissatisfaction, Depression, and Anxiety in Adolescents with Celiac Disease. *Front. Pediatr.* **2025**, *13*, doi:10.3389/fped.2025.1603009.
26. Daneshdoost, S.; Adams, K.; Cooper, M.; Timko, A.; Weaver, L.; Seidman, C.; Peebles, R.; Singh, A. Demystifying the Coexistence of Celiac Disease and Eating Disorders. *J Pediatr Gastroenterol Nutr* **2023**, *77*, S77.
27. Duerksen, D.; Jocelyn, S.; Weiten, D.; Green, K.; Duerksen, K.; Wilkey, K.; Walker, J.; Graff, L. Development of the Standardized Dietitian Integrated Evaluation Tool for Gluten-Free Diets (DIET-GFD): 2016 ACG Presidential Poster Award. *American Journal of Gastroenterology* **2016**, *11*, S461.
28. Fernández Miaja, M.; Díaz Martín, J.J.; Jiménez Treviño, S.; Suárez González, M.; Bousoño García, C. Study of Adherence to the Gluten-Free Diet in Coeliac Patients. *An. Pediatr. (Engl. Ed).* **2021**, *94*, 377–384, doi:10.1016/j.anpede.2020.06.012.
29. Fernández Miaja, M.; Suárez González, M.; Díaz Martín, J.J.; Jiménez Teviño, S.; Bousoño García, C.A. Analysis of Health-Related Quality Life in Celiac Patients. *Nutr. Hosp.* **2021**, *38*, 715–721, doi:10.20960/nh.03538.
30. Fishman, L.N.; Kearney, J.; Degroote, M.; Liu, E.; Arnold, J.; Weir, D.C. Creation of Experience-Based Celiac Benchmarks: The First Step in Pretransition Self-Management Assessment. *J. Pediatr. Gastroenterol. Nutr.* **2018**, *67*, e6–e10, doi:10.1097/MPG.0000000000001908.
31. Franco, L.; Nakano, E.Y.; Raposo, A.; Alturki, H.A.; Alarifi, S.N.; Chaves, C.; Teixeira-Lemos, E.; Romão, B. Eating Attitudes of Patients with Celiac Disease in Brazil: A Nationwide Assessment with the EAT-26 Instrument. *Nutrients* **2023**, *15*, 4796, doi:10.3390/nu15224796.
32. Fueyo-Díaz, R.; Magallón-Botaya, R.; Gascón-Santos, S.; Asensio-Martínez, Á.; Palacios-Navarro, G.; Sebastián-Domingo, J.J. Development and Validation of a Specific Self-Efficacy Scale in Adherence to a Gluten-Free Diet. *Front. Psychol.* **2018**, *9*, 299442, doi:10.3389/fpsyg.2018.00342.
33. Fueyo-Díaz, R.; Magallón-Botaya, R.; Sánchez-Calavera, M.A.; Asensio-Martínez, A.; Gascón-Santos, S. Protocol Development for a Scale to Assess Self-Efficacy in Adherence to a Gluten Free Diet: Self-Efficacy and Celiac Disease Scale. *Revista Española de Nutrición Humana y Dietética* **2015**, *19*, 160–166, doi:10.14306/renhyd.19.3.152.
34. Fueyo-Díaz, R.; Magallón-Botaya, R.; Gascón-Santos, S.; Asensio-Martínez, Á.; Palacios-Navarro, G.; Sebastián-Domingo, J.J. The Effect of Self-Efficacy Expectations in the Adherence to a Gluten Free Diet in Celiac Disease. *Psychol. Health* **2020**, *35*, 734–749, doi:10.1080/08870446.2019.1675658.
35. Fueyo-Díaz, R.; Montoro, M.; Magallón-Botaya, R.; Gascón-Santos, S.; Asensio-Martínez, Á.; Palacios-Navarro, G.; Sebastián-Domingo, J.J. Influence of Compliance to Diet and Self-Efficacy Expectation on Quality of Life in Patients with Celiac Disease in Spain. *Nutrients 2020, Vol. 12, Page 2672* **2020**, *12*, 2672, doi:10.3390/nu12092672.
36. Gaeta, F.; Valitutti, F.; Pisano, P.; Mandato, C. Gluten-Free Diet and Eating Disorders in Celiac Disease: An Open Question. *J. Pediatr. Gastroenterol. Nutr.* **2023**, *76*, 1174–1174, doi:10.1097/MPG.0000000000003823.
37. Garnweidner-Holme, L.; Sende, K.; Hellmann, M.; Henriksen, C.; Lundin, K.E.A.; Myhrstad, M.C.W.; Telle-Hansen, V.H. Experiences of Managing a Gluten-Free Diet on Multiple Levels of Society: A Qualitative Study. *BMC Nutr.* **2020**, *6*, doi:10.1186/s40795-020-00390-3.
38. Giannakopoulos, G.; Margoni, D.; Chouliaras, G.; Panayiotou, J.; Zellos, A.; Papadopoulou, A.; Liakopoulou, M.; Chrousos, G.; Kanaka-Gantenbein, C.; Kolaitis, G.; et al. Child and Parent Mental Health Problems in Pediatric Celiac Disease: A Prospective Study. *J. Pediatr. Gastroenterol. Nutr.* **2020**, *71*, 315–320, doi:10.1097/MPG.0000000000002769.
39. Grenet, P.; de Paillerets, F.; Gallet, J.P.; Babinet, J.M. Anorexic Forms of Celiac Disease. *J. Med. Chir. Prat.* **1975**, *146*, 254–259.
40. Grootenhuis, M.A.; Koopman, H.M.; Verrips, E.G.H.; Vogels, A.G.C.; Last, B.F. Health-Related Quality of Life Problems of Children Aged 8-11 Years with a Chronic Disease. *Dev. Neurorehabil.* **2007**, *10*, 27–33, doi:10.1080/13682820600691017.
41. Halmos, E.P.; Deng, M.; Knowles, S.R.; Sainsbury, K.; Mullan, B.; Tye-Din, J.A. Food Knowledge and Psychological State Predict Adherence to a Gluten-Free Diet in a Survey of 5310 Australians and New Zealanders with Coeliac Disease. *Aliment. Pharmacol. Ther.* **2018**, *48*, 78–86, doi:10.1111/apt.14791.

42. Hameed, S.; Sondhi, V. Experiences and Difficulties for Primary Caretakers of Children with Celiac Disease - A Qualitative Study. *Indian J. Gastroenterol.* **2023**, *42*, 791–799, doi:10.1007/s12664-023-01413-0.
43. Hoteit, M.; Chamas, Z.; Assaf, S.; Bouhairie, M.M.; Bahr, A.; Daccache, R.; Matar, R.; Hallal, M.; Hallal, M.; Hotayt, S.; et al. Nutritional Status, Nutrient Imbalances, Food-Related Behaviors and Dietary Supplements Use among Patients with Celiac Disease on a Gluten Free Diet in Lebanon: A National Cross-Sectional Study. *F1000Res.* **2022**, *11*, doi:10.12688/F1000RESEARCH.121859.3.
44. Joelson, A.M.; Geller, M.G.; Zylberberg, H.M.; Green, P.H.R.; Lebwohl, B. The Effect of Depressive Symptoms on the Association between Gluten-Free Diet Adherence and Symptoms in Celiac Disease: Analysis of a Patient Powered Research Network. *Nutrients 2018, Vol. 10, Page 538* **2018**, *10*, 538, doi:10.3390/nu10050538.
45. Kakkar, R.; Fung, A.; Barker, C.; Foster, A.; Hursh, B.E. The Experience of a Gluten-Free Diet in Children with Type 1 Diabetes and Celiac Disease. *J. Can. Assoc. Gastroenterol.* **2022**, *5*, 25–31, doi:10.1093/jcag/gwab013.
46. Kaltsa, M.; Garoufi, A.; Tsitsika, A.; Tsirogianni, A.; Papasteriades, C.; Kossiva, L. Patients with Eating Disorders Showed No Signs of Coeliac Disease before and after Nutritional Intervention. *Acta Paediatr.* **2015**, *104*, e319–e323, doi:10.1111/apa.12985.
47. Kalyoncu, T.; Çıldır, D.A. Trichotillomania in a Child with Celiac Disease: Irrespective of Iron Deficiency Anemia. *Psychiatry and Clinical Psychopharmacology* **2017**, *27*, 85–180, doi:10.1080/24750573.2017.1308709.
48. Karwautz, A.; Wagner, G.; Berger, G.; Sinnreich, U.; Grylli, V.; Huber, W.D. Eating Pathology in Adolescents with Celiac Disease. *Psychosomatics* **2008**, *49*, 399–406, doi:10.1176/appi.psy.49.5.399.
49. Khurana, B.; Lomash, A.; Khalil, S.; Bhattacharya, M.; Rajeshwari, K.; Kapoor, S. Evaluation of the Impact of Celiac Disease and Its Dietary Manipulation on Children and Their Caregivers. *Indian Journal of Gastroenterology 2015 34:2* **2015**, *34*, 112–116, doi:10.1007/s12664-015-0563-6.
50. Kinos, S.; Kurppa, K.; Ukkola, A.; Collin, P.; Lähdeaho, M.L.; Huhtala, H.; Kekkonen, L.; Mäki, M.; Kaukinen, K. Burden of Illness in Screen-Detected Children with Celiac Disease and Their Families. *J. Pediatr. Gastroenterol. Nutr.* **2012**, *55*, 412–416, doi:10.1097/MPG.0b013e31825f18ff.
51. Kinston, W.; Loader, P.; Miller, L. Talking to Families about Obesity: A Controlled Study. *Eat. Disord.* **1988**, 261–275.
52. Kujawowicz, K.; Mirończuk-Chodakowska, I.; Witkowska, A.M. Dietary Behavior and Risk of Orthorexia in Women with Celiac Disease. *Nutrients 2022, Vol. 14, Page 904* **2022**, *14*, 904, doi:10.3390/nu14040904.
53. Kurppa, K.; Lauronen, O.; Collin, P.; Ukkola, A.; Laurila, K.; Huhtala, H.; Mäki, M.; Kaukinen, K. Factors Associated with Dietary Adherence in Celiac Disease: A Nationwide Study. *Digestion* **2012**, *86*, 309–314, doi:10.1159/000341416.
54. Lecerf, J.M. Characteristics of Individuals Who Follow a Gluten-Free Diet. *Correspondances en MHND* **2022**, *26*, 7–7.
55. Leffler, D.A.; Dennis, M.; Edwards George, J.B.; Kelly, C.P. The Interaction between Eating Disorders and Celiac Disease: An Exploration of 10 Cases. *Eur. J. Gastroenterol. Hepatol.* **2007**, *19*, 251–255, doi:10.1097/MEG.0b013e328012db37.
56. Lu, Z.; Zhang, H.; Luoto, S.; Ren, X. Gluten-Free Living in China: The Characteristics, Food Choices and Difficulties in Following a Gluten-Free Diet – An Online Survey. *Appetite* **2018**, *127*, 242–248, doi:10.1016/j.appet.2018.05.007.
57. Maddison-Roberts, H.; Jones, C.; Satherley, R. OC65 Understanding the Psychological Experiences of Children and Young People with Coeliac Disease and Their Relationship with Food. *Frontline Gastroenterol.* **2024**, *15*, A45–A45, doi:10.1136/flgastro-2024-bspghan.62.
58. Nimel, M.; Jilowa, C.S.; Sharma, K.K.; Choudhary, O. Neuropsychiatric Manifestation of Celiac Disease: A Case-Control Study in North India. *Medical Journal of Dr. D.Y. Patil University* **2016**, *9*, 690–694, doi:10.4103/0975-2870.194183.
59. Marild, K.; Stordal, K.; Bulik, C.M.; Rewers, M.; Ekbom, A.; Liu, E.; Ludvigsson, J.F. Celiac Disease and Anorexia Nervosa: A Nationwide Study. *Pediatrics* **2017**, *139*, doi:10.1542/peds.2016-4367.
60. Marsilio, I.; Savarino, E.V.; Barberio, B.; Lorenzon, G.; Maniero, D.; Cingolani, L.; D'odorico, A.; D'incà, R.; Zingone, F. A Survey on Nutritional Knowledge in Coeliac Disease Compared to Inflammatory Bowel Diseases Patients and Healthy Subjects. *Nutrients 2020, Vol. 12, Page 1110* **2020**, *12*, 1110, doi:10.3390/nu12041110.

61. Mazzone, L.; Reale, L.; Spina, M.; Guarnera, M.; Lionetti, E.; Martorana, S.; Mazzone, D. Compliant Gluten-Free Children with Celiac Disease: An Evaluation of Psychological Distress. *BMC Pediatrics 2011 11:1* **2011**, *11*, 46-, doi:10.1186/1471-2431-11-46.
62. Meyer, S. Promoting Effective Self-Management of the Gluten-Free Diet: Children's and Adolescents' Self-Generated Do's and Don'ts. *International Journal of Environmental Research and Public Health 2022, Vol. 19, Page 14051* **2022**, *19*, 14051, doi:10.3390/ijerph192114051.
63. Meyer, S.; Lamash, L. Illness Identity in Adolescents With Celiac Disease. *J. Pediatr. Gastroenterol. Nutr.* **2021**, *72*, E42–E47, doi:10.1097/MPG.0000000000002946.
64. Meyer, S.; Monachesi, C.; Barchetti, M.; Lionetti, E.; Catassi, C. Cross-Cultural Participation in Food-Related Activities and Quality of Life among Children with Celiac Disease. *Children 2023, Vol. 10, Page 1300* **2023**, *10*, 1300, doi:10.3390/children10081300.
65. Meyer, S.; Rosenblum, S. Children With Celiac Disease: Health-Related Quality of Life and Leisure Participation. *Am. J. Occup. Ther.* **2016**, *70*, doi:10.5014/ajot.2016.020594.
66. Meyer, S.; Rosenblum, S. Development and Validation of the Celiac Disease-Children's Activities Report (CD-Chart) for Promoting Self-Management among Children and Adolescents. *Nutrients 2017, Vol. 9, Page 1130* **2017**, *9*, 1130, doi:10.3390/nu9101130.
67. Meyer, S.; Rosenblun, S.D. Diet Adherence and Daily Life Participation: Insights from Children and Adolescents with Coeliac Disease via a Standardised Scale. *Journal of Pediatric Gastroenterology and Nutrition - 50th ESPGHAN Annual Meeting* **2017**, *64*, 1–1017, doi:10.1097/01.mpg.0000516381.25680.b4.
68. Meyer, S.; Rosenblum, S. Daily Experiences and Challenges Among Children and Adolescents With Celiac Disease: Focus Group Results. *J. Pediatr. Gastroenterol. Nutr.* **2018**, *66*, 58–63, doi:10.1097/MPG.0000000000001635.
69. Meyer, S.; Rosenblum, S. Examining Core Self-Management Skills among Adolescents with Celiac Disease. *J. Health Psychol.* **2021**, *26*, 2592–2602, doi:10.1177/1359105320922304.
70. Meyer, S. Daily Activities, Participation and Quality of Life among Children and Adolescents with Celiac Disease. Dissertation, University of Haifa: Haifa, 2017.
71. Myléus, A.; Petersen, S.; Carlsson, A.; Hammarroth, S.; Högberg, L.; Ivarsson, A. Health-Related Quality of Life Is Not Impaired in Children with Undetected as Well as Diagnosed Celiac Disease: A Large Population Based Cross-Sectional Study. *BMC Public Health* **2014**, *14*, doi:10.1186/1471-2458-14-425.
72. Nayar, S.; Mahapatra, S.C. Nutritional Intake, Gluten-Free Diet Compliance and Quality of Life of Pediatric Patients with Celiac Disease. *Acta Hortic.* **2013**, *972*, 79–86, doi:10.17660/ActaHortic.2013.972.10.
73. Neis Elesbão, T.; Ridel Juzwiak, C. O Diagnóstico Da Doença Celíaca Como Ponto de Virada Nas Escolhas Alimentares e Novas Práticas Alimentares. *Sociedade e Cultura* **2024**, *27*, doi:10.5216/sec.v27.77467.
74. Nunes, F.; Almeida, J. Avoiding Reactions Outside the Home: Challenges, Strategies, and Opportunities to Enhance Dining out Experiences of People with Food Hypersensitivities. *Conference on Human Factors in Computing Systems - Proceedings* **2021**, doi:10.1145/3411764.3445662.
75. Okur-Ataş, Ş. Social-Emotional Competence, Executive Functions, and Diet Adherence in Children with Celiac Disease. *J. Health Psychol.* **2025**, doi:10.1177/13591053251349096.
76. Pancheva, R.; Dolinsek, J.; Panayotova, M.; Yankov, I.; Kofinova, D.; Nikolova, S.; Baycheva, M.; Georgieva, M. Bridging the Gap: Awareness, Knowledge, and Challenges of Living with Celiac Disease in Bulgaria. *Nutrients 2025, Vol. 17, Page 1267* **2025**, *17*, 1267, doi:10.3390/nu17071267.
77. Pozziani, G.; Casella, S.; Cattelan, C.; Righetto, C.; Pescarin, M.; Guariso, G. PP73 FROM CHILDHOOD TO ADOLESCENCE: THE COELIAC DISEASE EXPERIENCE FROM THE POINT OF VIEW OF THOSE WHO ARE GROWING UP AND THOSE WHO ARE TAKING CARE OF THEM. *Digestive and Liver Disease* **2011**, *43*, S439–S440, doi:10.1016/s1590-8658(11)60716-0.

78. Pratesi, C.P.; Häuser, W.; Uenishi, R.H.; Selleski, N.; Nakano, E.Y.; Gandolfi, L.; Pratesi, R.; Zandonadi, R.P. Quality of Life of Celiac Patients in Brazil: Questionnaire Translation, Cultural Adaptation and Validation. *Nutrients 2018, Vol. 10, Page 1167* **2018**, *10*, 1167, doi:10.3390/nu10091167.
79. Hallert, C.; Derefeldt, T. Mental Disorders in Adolescents With Celiac Disease. *Psychosomatics* **2004**, *45*, 325–335, doi:10.3109/00365528209181037.
80. Pynnönen, P.A.; Isometsä, E.T.; Verkasalo, M.A.; Kähkönen, S.A.; Sipilä, I.; Savilahti, E.; Aalberg, V.A. Gluten-Free Diet May Alleviate Depressive and Behavioural Symptoms in Adolescents with Coeliac Disease: A Prospective Follow-up Case-Series Study. *BMC Psychiatry 2005 5:1* **2005**, *5*, 14-, doi:10.1186/1471-244X-5-14.
81. Quick, V.M.; McWilliams, R.; Byrd-Bredbenner, C. Case–Control Study of Disturbed Eating Behaviors and Related Psychographic Characteristics in Young Adults with and without Diet-Related Chronic Health Conditions. *Eat. Behav.* **2012**, *13*, 207–213, doi:10.1016/j.eatbeh.2012.02.003.
82. Quick, V.M. Characteristics and Disturbed/Disordered Eating Behaviors of Young Adults with and without Diet-Related Chronic Health Conditions. **2011**, doi:10.7282/T3ZS2VTK.
83. Raber, C.L.; Coburn, S.S.; Pavone, L.; Stern, L.; Kerzner, B.; Badalyan, V. Tu2016 FEEDING AND EATING PROBLEMS IN CHILDREN WITH CELIAC DISEASE. *Gastroenterology* **2023**, *164*, S-1188, doi:10.1016/s0016-5085(23)03733-2.
84. Real Delor, R.E.; Aguilera, M.E. [Adherence Factors to the Gluten-Free Diet in People with Celiac Disease in Paraguay]. *Rev. Fac. Cien. Med. Univ. Nac. Cordoba* **2024**, *81*, 655–669, doi:10.31053/1853.0605.v81.n4.43220.
85. Emilio Real-Delor, R.; Elena Aguilera-Chamorro, M.; Asignada, E.; Judith Nava-González, E.; Celiaca, E. Facilidades y Barreras Para La Dieta Sin Gluten de Personas Con Enfermedad Celiaca Del Paraguay. *Revista Española de Nutrición Humana y Dietética* **2021**, *25*, 376–383, doi:10.14306/renhyd.25.4.1338.
86. Riehl, M.E.; Scarlata, K. Understanding Disordered Eating Risks in Patients with Gastrointestinal Conditions. *J. Acad. Nutr. Diet.* **2022**, *122*, 491–499, doi:10.1016/j.jand.2021.03.001.
87. Rimárová, K.; Dorko, E.; Diabelková, J.; Sulinová, Z.; Makovický, P.; Baková, J.; Uhrin, T.; Jenča, A.; Jenčová, J.; Petrášová, A.; et al. Compliance with Gluten-Free Diet in a Selected Group of Celiac Children in the Slovak Republic. *Cent. Eur. J. Public Health* **2018**, *26 Suppl*, S19–S24, doi:10.21101/cejph.a5369.
88. Roma, E.; Roubani, A.; Kolia, E.; Panayiotou, J.; Zellos, A.; Syriopoulou, V.P. Dietary Compliance and Life Style of Children with Coeliac Disease. *Journal of Human Nutrition and Dietetics* **2010**, *23*, 176–182, doi:10.1111/j.1365-277X.2009.01036.x.
89. Roos, S.; Hellström, I.; Hallert, C.; Wilhelmsson, S. Everyday Life for Women with Celiac Disease. *Gastroenterol. Nurs.* **2013**, *36*, 266–273, doi:10.1097/SGA.0b013e31829ed98d.
90. Russo, C.; Wolf, R.L.; Leichter, H.J.; Lee, A.R.; Reilly, N.R.; Zybert, P.; Green, P.H.R.; Lebwohl, B. Impact of a Child's Celiac Disease Diagnosis and Management on the Family. *Dig. Dis. Sci.* **2020**, *65*, 2959–2969, doi:10.1007/s10620-020-06316-0.
91. Sady, M.D.; Coburn, S.S.; Kramer, Z.; Streisand, R.; Kahn, I. Associations between Executive Functioning and Adherence in Pediatric Celiac Disease. *Child. Health Care* **2025**, *54*, 296–310, doi:10.1080/02739615.2023.2259291.
92. Sainsbury, K.; Halmos, E.P.; Knowles, S.; Mullan, B.; Tye-Din, J.A. Maintenance of a Gluten Free Diet in Coeliac Disease: The Roles of Self-Regulation, Habit, Psychological Resources, Motivation, Support, and Goal Priority. *Appetite* **2018**, *125*, 356–366, doi:10.1016/j.appet.2018.02.023.
93. Sainsbury, K.; Mullan, B.; Sharpe, L. Gluten Free Diet Adherence in Coeliac Disease. The Role of Psychological Symptoms in Bridging the Intention-Behaviour Gap. *Appetite* **2013**, *61*, 52–58, doi:10.1016/j.appet.2012.11.001.
94. Sainsbury, K.; Mullan, B.; Sharpe, L. Predicting Intention and Behaviour Following Participation in a Theory-Based Intervention to Improve Gluten Free Diet Adherence in Coeliac Disease. *Psychol. Health* **2015**, *30*, 1063–1074, doi:10.1080/08870446.2015.1022548.
95. Satherley, R.M.; Higgs, S.; Howard, R. Disordered Eating Patterns in Coeliac Disease: A Framework Analysis. *Journal of Human Nutrition and Dietetics* **2017**, *30*, 724–736, doi:10.1111/jhn.12475.

96. Satherley, R.M.; Howard, R.; Higgs, S. The Prevalence and Predictors of Disordered Eating in Women with Coeliac Disease. *Appetite* **2016**, *107*, 260–267, doi:10.1016/j.appet.2016.07.038.
97. Satherley, R.M. Disordered Eating Attitudes and Behaviours in Celiac Disease, University of Birmingham: Birminghan, 2017.
98. Schilling, K.W.; Yohannessen, K.; Araya, M.; Schilling, K.W.; Yohannessen, K.; Araya, M. Percepción de Estar Haciendo Bien La Dieta Sin Gluten y Adherencia al Tratamiento En Pacientes Pediátricos Con Enfermedad Celíaca. *Rev. Chil. Pediatr.* **2018**, *89*, 216–223, doi:10.4067/S0370-41062018000200216.
99. Schwantes, M. de C.; Maddison-Roberts, H.; Nakano, E.Y.; Botelho, R.B.A.; Zandonadi, R.P. Adaptation of the Child Coeliac Disease Food Attitudes and Behaviours Scale (Child CD-FAB) into Brazilian Portuguese: Translation and Evaluation of Reproducibility and Internal Consistency. *Nutrients* **2025**, *17*, 2704, doi:10.3390/nu17162704.
100. Shah, S.; Akbari, M.; Vanga, R.; Kelly, C.P.; Hansen, J.; Theethira, T.; Tariq, S.; Dennis, M.; Leffler, D.A. Patient Perception of Treatment Burden Is High in Celiac Disease Compared with Other Common Conditions. *Am. J. Gastroenterol.* **2014**, *109*, 1304–1311, doi:10.1038/ajg.2014.29.
101. Shani, M.; Kraft, L.; Müller, M.; Boehnke, K. The Potential Benefits of Camps for Children and Adolescents with Celiac Disease on Social Support, Illness Acceptance, and Health-Related Quality of Life. *J. Health Psychol.* **2022**, *27*, 1635–1645, doi:10.1177/1359105320968142.
102. Silva, L.C.; Nakano, E.Y.; Zandonadi, R.P. Division of Responsibility in Child Feeding and Eating Competence: A Cross-Sectional Study in a Sample of Caregivers of Brazilian Children with Celiac Disease. *Nutrients* **2024**, *16*, 1052, doi:10.3390/nu16071052.
103. Silva, L.C.; Nakano, E.Y.; Zandonadi, R.P. Eating Competence in Caregivers of Celiac Children: A Cross-Sectional Study Performed in Brazil. *Nutrition* **2024**, *119*, doi:10.1016/j.nut.2023.112326.
104. Duerksen, D.; Jocelyn, S.; Weiten, D.; Green, K.; Duerksen, K.; Wilkey, K.; Walker, J.; Graff, L. Development of the Standardized Dietitian Integrated Evaluation Tool for Gluten-Free Diets (DIET-GFD): 2016 ACG Presidential Poster Award: 1060. *Am. J. Gastroenterol.* **2016**, *111*, S461, doi:10.14309/00000434-201610001-01060.
105. Subramanian, L.; Coo, H.; Jane, A.; Flemming, J.A.; Acker, A.; Hoggan, B.; Griffiths, R.; Sehgal, A.; Mulder, D. Celiac Disease and Inflammatory Bowel Disease Are Associated With Increased Risk of Eating Disorders: An Ontario Health Administrative Database Study. *Clin. Transl. Gastroenterol.* **2024**, *15*, doi:10.14309/ctg.0000000000000700.
106. Terrone, G.; Parente, I.; Romano, A.; Auricchio, R.; Greco, L.; Del Giudice, E. The Pediatric Symptom Checklist as Screening Tool for Neurological and Psychosocial Problems in a Paediatric Cohort of Patients with Coeliac Disease. *Acta Paediatr.* **2013**, *102*, e325–e328, doi:10.1111/apa.12239.
107. Tittel, S.R.; Dunstheimer, D.; Hilgard, D.; Knauth, B.; Fröhlich-Reiterer, E.; Galler, A.; Wurm, M.; Holl, R.W. Coeliac Disease Is Associated with Depression in Children and Young Adults with Type 1 Diabetes: Results from a Multicentre Diabetes Registry. *Acta Diabetol.* **2021**, *58*, 623–631, doi:10.1007/s00592-020-01649-8.
108. Tokatly Latzer, I.; Rachmiel, M.; Zuckerman Levin, N.; Mazor-Aronovitch, K.; Landau, Z.; Ben-David, R.F.; GrafBar-El, C.; Gruber, N.; Levek, N.; Weiss, B.; et al. Increased Prevalence of Disordered Eating in the Dual Diagnosis of Type 1 Diabetes Mellitus and Celiac Disease. *Pediatr. Diabetes* **2018**, *19*, 749–755, doi:10.1111/pedi.12653.
109. Esenyel, S.; Ünal, F.; Vural, P. Depression and Anxiety in Child and Adolescents with Follow-up Celiac Disease and in Their Families. *Turk. J. Gastroenterol.* **2014**, *25*, 381–385, doi:10.5152/tjg.2014.4831.
110. Veen, M.; te Molder, H.; Gremmen, B.; van Woerkum, C. If You Can't Eat What You like, like What You Can: How Children with Coeliac Disease and Their Families Construct Dietary Restrictions as a Matter of Choice. *Sociol. Health Illn.* **2013**, *35*, 592–609, doi:10.1111/j.1467-9566.2012.01519.x.
111. Veen, M.; te Molder, H.; Gremmen, B.; van Woerkum, C. Quitting Is Not an Option: An Analysis of Online Diet Talk between Celiac Disease Patients. *Health N. Hav.* **2010**, *14*, 23–40, doi:10.1177/1363459309347478.

112. Wagner, G.; Zeiler, M.; Berger, G.; Huber, W.D.; Favaro, A.; Santonastaso, P.; Karwautz, A. Eating Disorders in Adolescents with Celiac Disease: Influence of Personality Characteristics and Coping. *Eur. Eat. Disord. Rev.* **2015**, *23*, 361–370, doi:10.1002/erv.2376.
113. Wagner, G.; Berger, G.; Sinnreich, U.; Grylli, V.; Schober, E.; Huber, W.D.; Karwautz, A. Quality of Life in Adolescents with Treated Coeliac Disease: Influence of Compliance and Age at Diagnosis. *J. Pediatr. Gastroenterol. Nutr.* **2008**, *47*, 555–561, doi:10.1097/MPG.0b013e31817fcb56.
114. Wagner, G.; Zeiler, M.; Grylli, V.; Berger, G.; Huber, W.D.; Woeber, C.; Rhind, C.; Karwautz, A. Coeliac Disease in Adolescence: Coping Strategies and Personality Factors Affecting Compliance with Gluten-Free Diet. *Appetite* **2016**, *101*, 55–61, doi:10.1016/j.appet.2016.02.155.
115. Wahab, R.J.; Beth, S.A.; Derks, I.P.M.; Jansen, P.W.; Moll, H.A.; Kiefte-De Jong, J.C. Celiac Disease Autoimmunity and Emotional and Behavioral Problems in Childhood. *Pediatrics* **2019**, *144*, doi:10.1542/peds.2018-3933.
116. Walsh, C.; Klassen, A.; Puran, A.; Hill, L.; Mehta, K.; Marcon M. What Matters to Children with Celiac Disease: A Qualitative Study Guiding the Development of the Celiac-Q Kids Patient-Reported Outcome Measure. *J Pediatr Gastroenterol* **2022**, *75*, S247–S248.
117. Weber, L.; Lugosi, P. The Event Experiences of Attendees with Food Allergies, Intolerances and Coeliac Disease: Risk Loaded Value-Creation/Destruction. *International Journal of Event and Festival Management* **2021**, *12*, 184–202, doi:10.1108/IJEFM-11-2020-0066.
118. Weisbrod, V.; Khavari, N.; Absah, I.; Lee, D.; Mallon, D.; Raber, C.; Badalyan, V.; Shull, M.; Verma, R.; Dunn, A.; et al. Gluten-free Schooling: Navigating Challenges and Triumphs for Children with Celiac Disease. *JPGN Rep.* **2025**, *6*, 99–106, doi:10.1002/jpr3.70013.
119. Welch, E.; Ghaderi, A.; Swenne, I. A Comparison of Clinical Characteristics between Adolescent Males and Females with Eating Disorders. *BMC Psychiatry* **2015**, *15*, doi:10.1186/s12888-015-0419-8.
120. Wolf, R.L.; Lebwohl, B.; Lee, A.R.; Zybert, P.; Reilly, N.R.; Cadenhead, J.; Amengual, C.; Green, P.H.R. Lower Quality of Life and Energy in Adults and Teenagers with Celiac Disease Are Associated with Greater Adherence and Knowledge about the Gluten-Free Diet. *Gastroenterology* **2017**, *152*, S159.
121. Wolf, R.L.; Lebwohl, B.; Lee, A.R.; Zybert, P.; Reilly, N.R.; Cadenhead, J.; Amengual, C.; Green, P.H.R. Correction to: Hypervigilance to a Gluten-Free Diet and Decreased Quality of Life in Teenagers and Adults with Celiac Disease (Digestive Diseases and Sciences, (2018), 63, 6, (1438-1448), 10.1007/S10620-018-4936-4). *Dig. Dis. Sci.* **2018**, *63*, 1982–1983, doi:10.1007/s10620-018-5049-9.
122. Xhakollari, V.; Canavari, M.; Osman, M. Why People Follow a Gluten-Free Diet? An Application of Health Behaviour Models. *Appetite* **2021**, *161*, doi:10.1016/j.appet.2021.105136.
123. Захарова, Р.В.П.И.П.; R.V.P.I.P.; Н.; Климов, И.Н.; Кочнева, Л.Я.; Гевандова, Л.Д.; Курьянинова, М.Г.; Стоян, В.А.; Кашников, М.В.; Иванова, В.С.; Ягупова, А.В.; Кашникова, А.В.; et al. Социальные Аспекты Соблюдения Безглютеновой Диеты Детьми и Подростками с Целиакией. *Российский вестник перинатологии и педиатрии* **2021**, *65*, 57–64, doi:10.21508/1027-4065-2020-65-6-57-64.
124. Zingone, F.; Massa, S.; Malamisura, B.; Pisano, P.; Ciacci, C. Coeliac Disease: Factors Affecting the Transition and a Practical Tool for the Transition to Adult Healthcare. *United European Gastroenterol. J.* **2018**, *6*, 1356–1362, doi:10.1177/2050640618787651.
125. Zysk, W.; Głąbska, D.; Guzek, D. Social and Emotional Fears and Worries Influencing the Quality of Life of Female Celiac Disease Patients Following a Gluten-Free Diet. *Nutrients 2018, Vol. 10, Page 1414* **2018**, *10*, 1414, doi:10.3390/nu10101414.
